# Self-consistent dispersal puts tight constraints on the spatiotemporal organization of species-rich metacommunities

**DOI:** 10.1073/pnas.2200390119

**Published:** 2022-06-21

**Authors:** Jonas Denk, Oskar Hallatschek

**Affiliations:** ^a^Department of Physics, University of California, Berkeley, CA 94720;; ^b^Department of Integrative Biology, University of California, Berkeley, CA 94720;; ^c^Peter Debye Institute for Soft Matter Physics, Leipzig University, 04103 Leipzig, Germany

**Keywords:** complex metacommunities, spatiotemporal abundance patterns, directed percolation

## Abstract

Biodiversity is often attributed to a dynamic equilibrium between the immigration and extinction of species. This equilibrium forms a common basis for studying ecosystem assembly from a static reservoir of migrants—the mainland. Yet, natural ecosystems often consist of many coupled communities (i.e., metacommunities), and migration occurs between these communities. The pool of migrants then depends on what is sustained in the ecosystem, which, in turn, depends on the dynamic migrant pool. This chicken-and-egg problem of survival and dispersal is poorly understood in communities of many competing species, except for the neutral case—the “unified neutral theory of biodiversity.” Employing spatiotemporal simulations and mean-field analyses, we show that self-consistent dispersal puts rather tight constraints on the dynamic migration–extinction equilibrium. When the number of species is large, species are pushed to the edge of their global extinction, even when competition is weak. As a consequence, the overall diversity is highly sensitive to perturbations in demographic parameters, including growth and dispersal rates. When dispersal is short range, the resulting spatiotemporal abundance patterns follow broad scale-free distributions that correspond to a directed percolation phase transition. The qualitative agreement of our results for short-range and long-range dispersal suggests that this self-organization process is a general property of species-rich metacommunities. Our study shows that self-sustaining metacommunities are highly sensitive to environmental change and provides insights into how biodiversity can be rescued and maintained.

## Significance

Dispersal can be critical to the maintenance of ecosystems as it allows local communities to be recolonized after extinction. However, it remains unclear whether the extinction-mitigating effect of dispersal persists when the number of competing species is large. Based on a spatially explicit mathematical description of metacommunities, we show that when many species coexist, each species operates near its extinction threshold, barely surviving due to dispersal. This has general consequences for spatiotemporal abundance patterns. For short-range dispersal, species organize into fractal spatiotemporal extinction patterns characteristic of a directed percolation phase transition. As species approach their extinction threshold, biodiversity is very sensitive to perturbation, suggesting that dispersal within a metacommunity puts tight constraints on the robustness and evolution of species-rich metacommunities.

The ecological dynamics of a community are shaped by the interplay of numerous factors, including inter- and intraspecies interactions, speciation, and species immigration. Finding meaningful theoretical models for the assembly and the stability of ecosystems is further complicated by the overwhelming number of species typically found in natural ecosystems (1–6). Despite this complexity, insights into some statistical properties of the ecosystem can be gained by assuming a dynamic equilibrium between extinction of species in a local community (island) and immigration of species from some static reservoir (mainland)—a concept that builds on MacArthur and Wilson’s (7) classical theory of island biography. For instance, assuming that, regardless of species identity, individuals have the same rates of reproduction and death [neutrality assumption (8, 9)], one can analytically derive the static abundance distribution based on the balance between extinction and the continuous emergence of new species (speciation) (10–14). While the neutrality assumption seems to be a crude simplification of natural ecosystems and has generated much controversy (15–20), its predicted abundance distributions are in surprisingly good agreement with the typical log series–and log normal–like distributions observed across different ecological systems (21–24). Embracing differences among species, Robert May (25, 26) proposed in his seminal work that the diversity of an ecosystem becomes unstable when the ecosystem is too complex because the number of species, their connectivity, or differences in species interactions become too large. Motivated by May’s work, theoretical studies based on random-interaction models (27–32) have found that increasing differences in competitive interspecies interactions can destabilize a community on an island and lead to strong temporal fluctuations in the species’ abundances (30, 32–34), consistent with recent microbialecology experiments (35, 36)

While in mainland–island models, the dynamic equilibrium of a local community strongly depends on migrants from a static mainland, it is natural to ask how biodiversity can be maintained when migrants instead come from other local communities themselves. Natural ecosystems, for instance, are often better represented as metacommunities composed of many coupled communities, between which individuals disperse (37, 38). This imposes an underlying self-consistency of dispersal in metacommunities: The pool of migrants is determined by what is sustained in the ecosystem, which in turn, depends on the migrant pool. Mainland–island models generally lack this self-consistency because there dispersal from the mainland is assumed to be a free parameter that is independent of the population on the island.

Over the past five decades, theoretical (34, 39–48) and experimental studies (49–53) on metacommunities and metapopulations have repeatedly shown that dispersal between patches can alleviate global extinctions of species and stabilize biodiversity. Simply put, dispersal can prevent global extinction because even if species go extinct on some patches, they can still be present on other patches and from there, recolonize the patches where they had gone extinct. This vital role of dispersal becomes most evident in patch occupancy models (37–40, 54, 55), which lack population dynamics on patches, and where the colonization rate must be sufficiently large to avert species extinction. When incorporating population dynamics on patches, at least for metapopulations, the self-consistency constraint of dispersal has proven useful in deriving the equilibrium distribution of individuals on a patch (43, 44, 56, 57) and a minimal dispersal rate necessary to avoid extinction (43, 56). However, especially when many species interact in a metacommunity, the effects of dispersal on biodiversity and the spatiotemporal abundance patterns of species are much less clear and depend on the underlying population dynamics (34, 45, 47, 48, 57, 58). For instance, when local extinctions are driven by large oscillations in the species’ abundances (e.g., through predator–prey interactions), the interplay between dispersal and local extinction can lead to intriguing spatiotemporal abundance patterns, including spiral waves (58) and—when the number of species is large—chaotic dynamics (34, 47).

Here, we focus on an alternative regime, where interspecies competition is weak (relative to intraspecies competition) and the species’ dynamics due to their interactions are overshadowed by demographic fluctuations. Weak interspecies competitions may, for instance, occur when species occupy different niches (59, 60), as proposed for various natural ecosystems (61–65). Assuming a scenario where species compete only weakly, how, if at all, do these interactions then affect the macroscopic properties of the metacommunity? Can we make general statements about spatiotemporal abundance patterns when the number of competing species is large? And, based on the results for such a “weak competition” model, can we draw conclusions for more complex interaction structures? To address these questions, we develop a stochastic discrete diffusion model of species-rich metacommunities. For a metacommunity with weak interspecies competition, we find that, as species numbers increase, local demographic fluctuations within species increase and drive the system to a dispersal-dependent edge of global extinction. Motivated by the large variation of dispersal length scales in natural ecosystems, we consider dispersal on two limiting length scales: short-range dispersal between nearest neighboring patches and spatially uniform dispersal between all patches (global dispersal). For short-range dispersal, we find that the proximity of species to their critical extinction threshold results in fractal spatiotemporal patterns that fall into the universality class of directed percolation. For global dispersal, we derive an analytical mean-field approximation for the abundance distribution, which resembles distributions commonly observed in natural ecological systems. Finally, we discuss the relevance of our results for various generalizations of our mathematical description and applications to empirical studies of natural ecological system. Our study sheds light on spatially structured metacommunities and suggests that self-consistent dispersal renders a species-rich metacommunity much more sensitive to perturbations, including environmental change, than previously thought.

## Results

### Lotka–Volterra Model of Metacommunities with Weak Interspecies Competition.

**Fig. 1.**
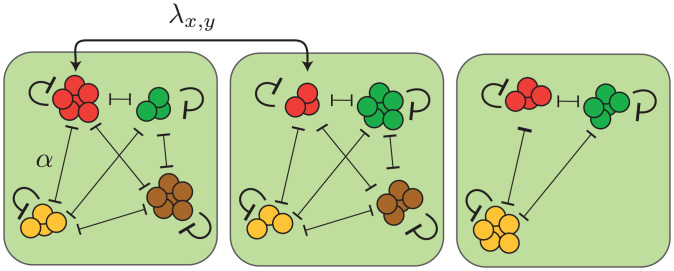
Metacommunity framework with niche interactions. Populations of different species (circles illustrate individuals) grow on patches (green) with growth rate *r* and carrying capacity *K*. Competition between species, characterized by the competition strength *α*, is weaker than self-limitation (e.g., due to limited niche overlap), allowing multiple species to coexist on a patch. Furthermore, individuals disperse between different patches *x* and *y* at a dispersal rate $\lambda_{x,y}$. Especially when a species’ population size on a patch is low (e.g., due to a large number of competitors), demographic fluctuations promote stochastic extinctions of species on individual patches (extinction of the brown species on the right patch.

In the following, we consider *S* species that live in a metacommunity of *P* coupled communities (patches), where *P* is assumed to be large. Lotka–Volterra equations provide an intuitive and simple way to take into account self-limiting interactions and interactions between species (66). The dynamics of the species’ populations are modeled by the following set of generalized Lotka–Volterra equations (see Fig. 1 for a graphical representation):

[1] $$\begin{array}{l} \partial_{t}N_{x,i}(t)=rN_{x,i}\left(1-\frac{\alpha }{K}\sum_{j,j\neq i}^{S}N_{x,j}-\frac{N_{x,i}}{K}\right)\\ \;+\sum_{y}^{P}\lambda_{y,x}(N_{y,i}-N_{x,i})+\sqrt{N_{x,i}}\;\eta , \end{array}$$

where $N_{x,i}$ denotes the abundance of species i∈{1,…S} on the patch x∈{1,…,P}. The first term in Eq. **1** describes growth of a species’ population at a growth rate *r*, which is bounded by self-limiting interactions within a species as well as competition with all other species on the same patch. For a clearer presentation of our main results, the strengths of interspecies interactions are chosen to be identical for all species and set to *α*. Later, we relax this assumption and will allow variations in the species’ interspecies interactions, growth rates, and dispersal rates. In the absence of interspecies interactions (i.e., *α* = 0), self-limiting interactions lead to population saturation at a carrying capacity *K*. Thus, *α* can be interpreted as the ratio of interspecies and self-limiting interaction strengths. By setting 0<α<1, we assume that self-limiting interactions are stronger than competition between species. This assumption emulates ecosystems where species coexist by occupying different niches (59, 60), as frequently suggested for natural microbial ecosystems (61–65) [*α* could be interpreted as a measure for the niche overlap (66, 67)]. In the following, we focus on weak competition and choose 0<α≪1, which will allow multiple species to coexist on each patch. On the other hand, strong interspecies competition (α>1) is known to promote exclusion between species (32, 66). The special case *α* = 1 marks the neutral scenario (8) and sets the boundary between niche partitioning (0<α<1) and competitive exclusion (α>1). The second term in Eq. **1** takes into account dispersal, where $\lambda_{x,y}$ denotes the dispersal rate between two patches *x* and *y* and is assumed equal for all species (we will later relax the assumption of equal dispersal rates). The last term in Eq. **1** reflects demographic fluctuations due to random births and deaths of individuals within a population, where $\eta_{x,i}$ denotes uncorrelated noise with zero mean and variance $\omega^{2}$. The square root dependence of demographic noise on the density ensures that the expected variance of fluctuations is proportional to the expected number of birth or death events during one generation and has been derived in various contexts from discrete descriptions of growing populations (68–70).

Assuming 0<α<1, the deterministic dynamics of Eq. **1** (i.e., ignoring noise) possess a stable solution in which all species coexist on all patches at equal abundance $N^{*}$ with

[2] $$N^{*}=K/[1+\alpha (S-1)].$$

The inverse dependence of $N^{*}$ on the number of species *S* suggests that when the number of species is large, the population size of each species on a patch can become very small, favoring (local) stochastic extinctions of species (compare with the extinction of brown species illustrated in Fig. 1). This leads us to suspect that, especially in the case of many coexisting species, dispersal plays an important role in offsetting local species extinctions. Based on our metacommunity model, Eq. **1**, we can now investigate the role of dispersal for metacommunities of weakly competing species and ask how the balance of dispersal and stochastic extinctions shapes spatiotemporal abundance patterns in a species-rich metacommunity. In the following sections, we will address this question for the two limiting scenarios of short-range dispersal and uniform dispersal between all patches (global dispersal). Our results from the fully symmetric case of indistinguishable species, Eq. **1**, will provide insights that contribute significantly to the understanding of species-rich metacommunities with more general properties, which we will discuss in the final section.

### The Dispersal Rate Needs to Exceed a Threshold to Prevent Global Extinction.

First, we consider dispersal on the smallest length scale where individuals can disperse only between neighboring patches. This assumption has, for instance, been extensively applied in studies of expanding microbial biofilms (71–74). To implement short-range dispersal in one dimension, we assume a one-dimensional lattice of patches and set $\lambda_{x,y}=(1/2)\lambda$ for all pairs of neighboring patches *x* and *y* and $\lambda_{x,y}=0$ otherwise. The dispersal term in Eq. **1** then reduces to $\left(1/2)\lambda (N_{x+1,i}+N_{x-1,i}-2N_{x,i}\right)$, where we furthermore assume periodic boundary conditions. First, we fix *r*, *K*, and *α* (with α≪1) and vary the dispersal rate *λ* for different numbers of species *S*.

**Fig. 2.**
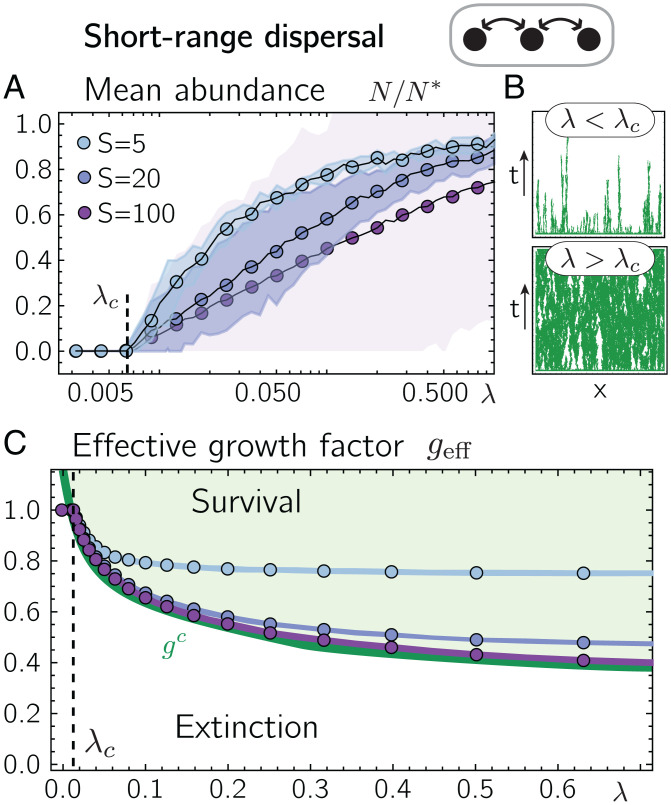
Dispersal–extinction balance and self-organization to the extinction threshold. (*A*) Numerical solutions of Eq. **1** for short-range dispersal show that for dispersal rates *λ* below *λ_c_*, all species go globally extinct, whereas for larger dispersal rates, the mean population size, *N* (circles), assumes nonzero values. Shaded areas denote standard deviations of patch-averaged abundances *N_i_* across all species. (*B*) Spatiotemporal dynamics of a representative species for dispersal rates below and above the threshold *λ_c_* for *S* = 5. Axes denote time *t* (measured in units of $\omega^{-1}$) and the location (patch) *x*. Regions where the species is present and extinct are colored in green and white, respectively. (*C*) For dispersal rates *λ* larger than *λ_c_*, the mean effective growth factor $g_{\text{eff}}={\left(PS\right)}^{-1}\sum_{x,i}g_{\text{eff}}^{\left(x,i\right)}$ (circles) drops below one. The shaded areas denote the standard deviation of the patch-averaged effective growth factors $g^{\left(i\right)}$ across all species (which is virtually zero). For increasing *S*, the effective growth factors asymptotically approach the single-species threshold value *g^c^* (green solid line). The green and white shaded areas indicate parameter regimes where the single-species dynamics, Eq. **4**, yield finite and zero population sizes, respectively. Parameter values are r=0.3, K=10, α=0.1, and *P* = 500. As an initial condition, we chose $N_{x,i}=K$ for all patches and species with small random perturbations.

When numerically solving the dynamics in Eq. **1** with short-range dispersal (for details on the numerical solution, see *SI Appendix*, section 1), we find that for zero and small dispersal rates *λ*, all species eventually go extinct due to demographic fluctuations. In contrast, when *λ* exceeds a critical threshold value *λ_c_*, the average population size $N={\left(PS\right)}^{-1}\sum_{x,i}N_{x,i}$ after the final time step of our numerical solution is finite and increases with *λ* (circles in Fig. 2*A*). Here, species occasionally go extinct on individual patches but are able to recolonize these patches eventually (Fig. 2*B*).

Close above *λ_c_*, the species’ mean population sizes are infinitesimal small so that interactions between them should be negligible. Consistently, we find that the critical dispersal rate *λ_c_* does not depend on the competition strength *α* nor the number of species *S* (Fig. 2*A*). Equations of the form of Eq. **1** without interspecies interactions (i.e., *α* = 0) are well studied in the context of the directed percolation (see refs. 75 and 76 for reviews). In particular, for one-species metacommunities (i.e., metapopulations), the interplay of population growth, dispersal, and demographic fluctuations is known to lead to a nonequilibrium phase transition from a phase of zero population size (absorbing phase) to a phase of finite population sizes (active phase) marking the directed percolation threshold. In our multispecies metacommunity, we thus recover the directed percolation threshold at the critical dispersal rate *λ_c_*, independent of the interaction strength *α* and the number of interacting species *S*.

Comparing the patch-averaged population sizes of individual species, $N_{i}=P^{-1}\sum_{x}N_{x,i}$, after the last time step of our numerical solutions, we find that these can strongly differ across species, especially when the number of species is large (compare with the shaded areas in Fig. 2*A*). In particular, some species occupy only a very small fraction of patches. Some species also die out globally even for $\lambda >\lambda_{c}$, which we attribute to the finite number of patches in our numerical solution.

### Species Packing Pushes Growth Rates toward the Extinction Threshold.

Motivated by the wide variation in population size across species, in the following we aim to better understand the metacommunity dynamics on the individual species level. To investigate the dynamics of individual species, we first rewrite the deterministic growth dynamics of a species *i* on patch *x* (the first term in Eq. **1**) as

[3] $$\partial_{t}N_{x,i}(t)=rN_{x,i}\left[g_{\text{eff}}^{\left(x,i\right)}-N_{x,i}/K\right]\text{​}.$$

Here, we defined the effective growth factor $g_{\text{eff}}^{\left(x,i\right)}≔1-(\alpha /K)\sum_{j,j\neq i}^{S}N_{x,j}$ of species *i* on patch *x*, which can be understood as the ratio of the species’ growth rate in the presence of competing species and its growth rate in the absence of competing species (i.e., *r*). Depending on the degree to which interspecies competition suppresses population growth, the effective growth factor thus takes on values less than or equal to one. Eq. **3** suggests that—in the absence of dispersal and demographic fluctuations—a species’ population will grow and assume a finite population size precisely if its effective growth factor is larger than zero. Ignoring demographic fluctuations, this observation has been used to derive expressions for the abundance distributions in well-mixed species-rich communities with small constant immigration when interspecies interactions are randomly distributed (28, 29, 31).

In our numerical solutions of Eq. **1**, we observe that the patch-averaged effective growth factors of a species, $g_{\text{eff}}^{\left(i\right)}=P^{-1}\sum_{x,i}g_{\text{eff}}^{\left(x,i\right)}$, are virtually identical across different species (Fig. 2*C*; see *SI Appendix*, section 2 for their distribution). We find that for dispersal rates *λ* above the critical dispersal rate *λ_c_*, the patch-averaged effective growth factors $g_{\text{eff}}^{\left(i\right)}$ drop to values below one, consistent with the fact that there, species coexist and thereby, suppress each other’s growth through competition. Furthermore, when the number of species *S* increases, $g_{\text{eff}}^{\left(i\right)}$ decreases until it eventually saturates at a positive finite value. Why does the patch-averaged effective growth factor saturate for large *S*, and what is the role of this saturation for the spatiotemporal dynamics of the metacommunity?

To better understand the role of the effective growth factor $g_{\text{eff}}^{\left(x,i\right)}$ in the metacommunity’s dynamics, we substitute $g_{\text{eff}}^{\left(x,i\right)}$ in Eq. **3** for a fixed parameter *g* and numerically solve the dynamics of the metacommunity for different choices of *g* and *λ*, including demographic fluctuations and dispersal. Species are thus no longer coupled with each other, and the dynamics of every species’ population size *N_x_* on a patch *x* reduce to

[4] $$\begin{array}{l} \partial_{t}N_{x}(t)=N_{x}r\left[g-\frac{N_{x}}{K}\right]\\ +\frac{1}{2}\lambda (N_{x+1}+N_{x-1}-2N_{x})+\sqrt{N_{x}}\;\eta . \end{array}$$

From directed percolation theory (75, 76), we expect Eq. **4** to feature a transition from a state of zero population size to a state of finite population sizes depending on the parameters *r*, *g*, *K*, and *λ*. Indeed, solving the one-species dynamics Eq. **4** numerically for different *g* and fixed *r*, *K*, and *λ*, we identify a threshold value for *g*, which we denote *g^c^* (the green line in Fig. 2*C*). When *g* is smaller than *g^c^*, the dynamics (Eq. **4**) eventually lead to stochastic extinction, while for *g* larger than *g^c^*, the dynamics (Eq. **4**) lead to finite population sizes (regimes “Survival” and “Extinction” in Fig. 2*C*, respectively). Interestingly, we find that this threshold value $g^{c}(r,K,\lambda )$ marks the asymptotic values of the patch-averaged effective growth factors $g_{\text{eff}}^{\left(i\right)}$ in the metacommunity for large *S*:

[5] $$g_{\text{eff}}^{\left(i\right)}(\lambda ,S)\overset{S\gg 1}{\to }g^{c}(\lambda ).$$

Hence, when the number of competing species is large, the patch-averaged effective growth factor of each species is pushed toward the critical threshold value, at which the species’ growth is just strong enough to balance stochastic extinctions. This suggests that every species operates close to its extinction threshold (percolation threshold). Importantly, this self-organization is not restricted to a particular choice of the dispersal rate or the remaining model parameters r, K, and *α* but occurs for any $\lambda >\lambda_{c}$ when the number of species *S* is large. In *SI Appendix*, section 3, we systematically increase the interaction strengths and argue that the observed self-organization toward the critical extinction threshold, Eq. **5**, is present as long as the number of coexisting species at each patch (local diversity) is much greater than one, which is especially the case for weak species interactions.

**Fig. 3.**
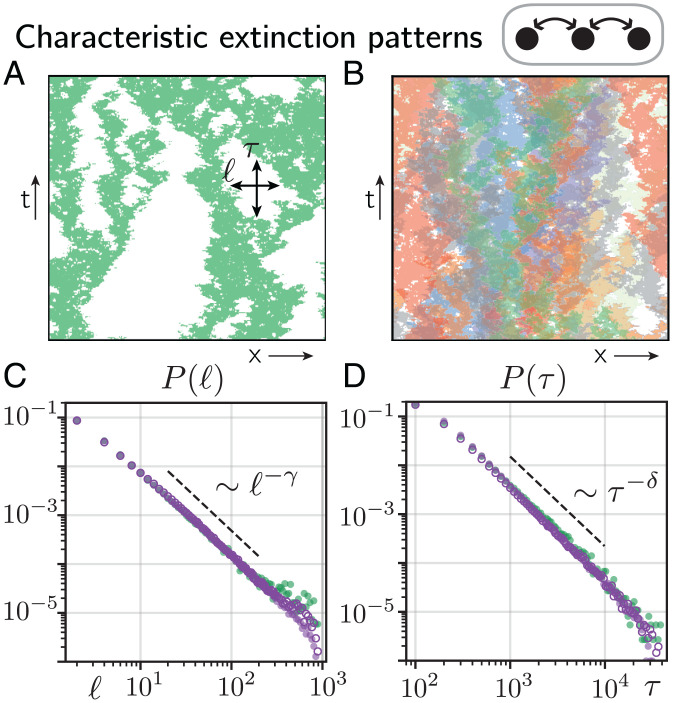
Characteristic spatiotemporal patterns in species-rich metacommunities. (*A*) For dispersal rates larger than *λ_c_* (here, λ=0.89), the spatiotemporal dynamics of a species in a species-rich metacommunity (*S* = 100) show extinction patterns of various lengths l and times *τ* that can range up to the system size and the time of our numerical solution, respectively. Two hundred time steps (generations) between generation 10^4^ and $5\times 10^{4}$ are shown. (*B*) Spatiotemporal dynamics of nine randomly picked species for parameters as in *A*. (*C* and *D*) The distributions P(l) and P(τ) of extinction lengths l and times *τ* (indicated in *A*), respectively. Purple open and closed circles denote the distributions for *S* = 100 with small and large dispersal rates (λ=0.32 and λ=0.89, both larger than *λ_c_*), respectively, and green circles show the distribution for the single-species dynamics, Eq. **4**, at criticality $g≳g^{c}$ [*g* is set to $S^{-1}\sum_{i}g_{\text{eff}}^{\left(i\right)}$ measured in a metacommunity with *S* = 100]. Dashed black lines indicate power-law distributions with exponents γ=−1.747 and δ=−1.840, respectively, as predicted from directed percolation theory. Parameter values are r=0.3, K=10, α=0.1, and P=1,000. As an initial condition, we chose $N_{x,i}=K$ for all patches x∈{1,…P} and species i∈{1,…S} with small random perturbations.

From a physical perspective, we expect that close to a critical transition, the characteristic timescale and length scale of the system’s dynamics diverge, and the system’s observables obey scaling laws that are—to some extent—independent of model details, such as microscopic interaction assumptions (75–77). Indeed, when the number of species is large, our numerical solutions display spatiotemporal extinction patterns whose length scale and timescale extend to scales comparable with the system size and simulation time of our numerical solution, respectively (Fig. 3 *A* and *B*). More precisely, we find that the length l of connected regions in which individual species are extinct is well approximated by a power-law distribution (Fig. 3*C*, purple circles). Similarly, the time *τ* between a species’ extinction on a patch and its successful recolonization of that patch from adjacent patches follows a power-law distribution (Fig. 3*D*, purple circles). Our above analyses indicate that if the number of species in the metacommunity is large, each species’ dynamics follow the dynamics of a species that is uncoupled from other species but has fixed growth parameters close to the percolation threshold. To further test this hypothesis, we numerically solved the single-species dynamics, Eq. **4**, for values of *g* close to *g_c_* [for instance, when we choose $g=S^{-1}\sum_{i}g_{\text{eff}}^{\left(i\right)}$ for *S* = 100]. We find that the distribution of extinction lengths and times follows power laws that are in excellent agreement with the distribution we found for the species-rich metacommunity (compare green and purple circles in Fig. 3 *C* and *D*). Furthermore, these power laws are well described by exponents found in one-dimensional directed percolation of a single species (78, 79) (dashed lines in Fig. 3 *C* and *D*; for a more detailed discussion of the observed power-law exponents, see *SI Appendix*, section 4). Together, our results strongly suggest that in the species-rich metacommunity, each species follows the dynamics of a species that is uncoupled from other species with fixed growth parameters close to the percolation threshold. The observed convergence of the single-species dynamics to the universality class of directed percolation underscores the relevance of our results for generalizations of our theoretical description and suggests a broad applicability to natural ecosystems (75, 76) (see *Discussion*).

While the effective growth factors averaged over patches are driven toward the threshold *g^c^* for all species, the effective growth factors can differ between different patches (*SI Appendix*, section 2). Specifically, we find that species experience effective growth factors $g_{\text{eff}}^{\left(x,i\right)}$ that lie below the threshold *g^c^* on some patches. Our results thus show that species can survive effective growth factors below the threshold *g^c^* on some patches as long as these patches are balanced by patches with effective growth rates above the extinction threshold, such that the patch-averaged effective growth factor of a species exceeds the extinction threshold.

As exemplified in various metacommunities, including microbial (80, 81) and plant communities (82, 83), different length scales of dispersal can confer very different statistical properties to an ecosystem, with important consequences on the evolutionary dynamics of the community. In the next section, we will, therefore, explore the question of whether and in what ways our results for short-range dispersal apply to larger length scales of dispersal.

### Species-Rich Metacommunities with Global Dispersal.

So far, we have studied how a metacommunity can maintain itself if we assume dispersal between nearest neighboring patches. We now explore metacommunity dynamics under the opposite dispersal pattern of global, all to all dispersal. This dispersal pattern allows us to 1) check how sensitive our main results are to dispersal patterns and 2) obtain concrete analytical results.

Specifically, we assume that all patches are connected through dispersal with a dispersal rate $\lambda_{x,y}=\lambda /P$. The dispersal term in Eq. **1** then reduces to $\lambda ({\bar{N}}_{i}-N_{x,i})$, where ${\bar{N}}_{i}$ denotes the abundance of species *i* averaged over all *P* patches. For our analytical mean-field approach, we first express the interaction term Eq. **1** through the species-averaged abundance on a patch defined as ${\hat{N}}_{x}=S^{-1}\sum_{i}N_{x,i}$. Then, by treating the mean fields ${\hat{N}}_{x}$ and ${\bar{N}}_{i}$ as deterministic mean-field parameters, we can map the dynamics in Eq. **1** to the solvable problem of a Brownian particle in a fixed potential. Since in our basic model (Eq. **1**), all species are indistinguishable, the mean abundances ${\bar{N}}_{i}$ and ${\hat{N}}_{x}$ are equal in equilibrium (in the limit of an infinite number of species and patches). Finally, we can derive an analytic expression for the abundance distribution as a function of the mean species abundance $\bar{N}≔{\bar{N}}_{i}={\hat{N}}_{x}$ and the control parameters r, K, α, S, and *λ* (for a detailed derivation, see *SI Appendix*, section 5). The abundance distribution is given by

[6] $$P[N,\bar{N},r,K,\lambda ]=\frac{1}{Z}\frac{1}{N^{1-2\lambda \bar{N}}}{\text{e}}^{-Kr{\left[\left(g_{\text{eff}}-\lambda /r\right)-\frac{N}{K}\right]}^{2}},$$

where *Z* denotes the normalization constant, and we defined the mean-field effective growth factor $g_{\text{eff}}≔1-(\alpha /K)(S-1)\bar{N}$. We can now solve for the mean abundance $\bar{N}$ self-consistently by calculating the statistical mean abundance based on the distribution (Eq. **6**), ${\langle N\rangle }_{P}$, and demanding that $\bar{N}={\langle N\rangle }_{P}$. Eventually, this yields a closed form of the species abundance distribution. From the species abundance distribution, we can then calculate various equilibrium quantities, such as the mean-field effective growth factor and the mean local diversity (*SI Appendix*, sections 5 and 6). Depending on the choice of parameters, the abundance distribution P approaches forms that have been commonly found in natural ecosystems (13, 84–87) and mathematically derived from previous ecological models (10–14, 88, 89). For instance, when the dispersal rate is small (i.e., $\lambda \bar{N}\ll 1$), Eq. **6** follows the scaling $P[N]\propto x^{N}/N$, which is commonly referred to as Fisher log series and denotes one of the most widely used abundance distributions in ecology (see refs. 90 and 91 for reviews). For larger dispersal rates, Eq. **6** suggests a Gaussian contribution with a maximum at $N=K(g_{\text{eff}}-\lambda /r)$ and a variance K/(2r) (see *SI Appendix*, section 5 for details).

Similar to short-range dispersal (compare with Fig. 2*A*), we find that the mean abundance of all species undergoes a bifurcation at a critical dispersal rate *λ_c_* from zero to nonzero values (*SI Appendix*, section 5). For Kλ≪Kr, the critical dispersal rate can be approximated by

[7] $$\lambda_{c}(r,K)\approx {\text{e}}^{-Kr}\sqrt{\frac{r}{4\pi K}},$$

which is independent of the number of interacting species and the interaction parameter *α*. In the limiting case of Kr≪Kλ, we obtain the approximation

[8] $$\lambda_{c}(r,K)\approx \frac{1}{2K}-r.$$

**Fig. 4.**
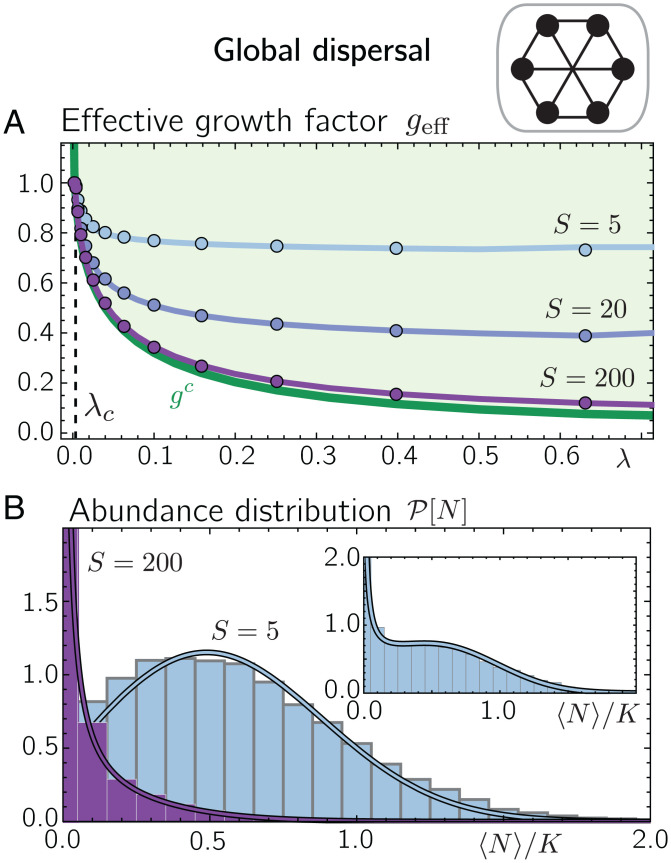
Mean-field approach is in good agreement with numerical simulations for global dispersal. (*A*) For dispersal rates *λ* larger than *λ_c_*, the mean effective growth factor $g_{\text{eff}}$ drops below one (circles and solid lines show numerical solutions and mean-field solutions for $g_{\text{eff}}$, respectively). For large *S*, the mean effective growth factor asymptotically approaches the single-species threshold value *g^c^* (green solid line; calculated from mean-field theory). (*B*) Abundance distributions for *S* = 5 (light blue) and *S* = 200 (purple) for λ=0.1 (main plot) and λ=0.04 (*Inset*). Histograms display the numerical solutions with global dispersal, and solid lines show the corresponding mean-field solutions. Parameter values are r=0.3, K=10, and α=0.1. For the numerical solutions, we chose initial conditions $N_{x,i}=K$ for all patches x∈{1,…P} with *P* = 500 and species i∈{1,…S} with small random perturbations.

Both limiting behaviors, Eqs. **7** and **8**, are in very good agreement with numerical solutions of the metacommunity dynamics (*SI Appendix*, section 5). The observation of a finite dispersal threshold for global dispersal is consistent with previous studies of metapopulations (43, 44, 56), which considered global dispersal through a shared reservoir. When the number of species *S* increases, we find that the mean effective growth factor $g_{\text{eff}}$ asymptotically approaches the single-species threshold value $g^{c}(\lambda )$ (solid lines in Fig. 4*A*; for the mean-field solution of *g^c^* and its limiting behaviors for small and large dispersal rates, see *SI Appendix*, section 6). Similar to short-range dispersal, this suggests that species in the species-rich metacommunity operate close to their extinction threshold.

Treating ${\bar{N}}_{i}$ and ${\hat{N}}_{x}$ as deterministic mean fields is based on the assumption that the number of patches *P* and the number of species per patch are large enough such that fluctuations in ${\bar{N}}_{i}$ and ${\hat{N}}_{x}$ across species and patches, respectively, are negligible. While *P* can simply be chosen large in our numerical solutions, the diversity per patch depends on the model parameters, including the competition strength *α*. When the diversity per patch is much larger than one, such as for weak competition (0<α≪1), as is the main focus of this work, we find very good agreement between our mean-field predictions and our numerical solutions (compare Fig. 4*A* with Fig. 4*B*). However, for larger *α*, especially α≲1, the diversity per patch can drop to only one species, and we observe deviations between our mean-field and numerical solutions (for a more detailed discussion of limitations of our mean-field theory, see *SI Appendix*, section 7).

### Variation in Growth Parameters Drives the Extinction of a Part of the Community.

The proximity of species to extinction in a species-rich metacommunity allows several implications about the sensitivity of the metacommunity to perturbations. For instance, in a species-rich metacommunity, even a small variation in the dispersal rates or growth rates between species may lift the effective growth factors of some species below the extinction threshold and thereby, lead to their global extinction. To investigate the effect of differences in the species’ growth dynamics and dispersal, we generalize Eq. **1** and consider the following dynamics in the metacommunity:

[9] $$\begin{array}{l} \partial_{t}N_{x,i}(t)=r_{i}N_{x,i}\left(1-\frac{N_{x,i}}{K}\right)-r\sum_{j,j\neq i}^{S}\frac{\alpha_{i,j}}{K}N_{x,j}\\ \;+\sum_{y}^{P}\lambda_{i,y,x}(N_{y,i}-N_{x,i})+\sqrt{N_{x,i}}\;\eta , \end{array}$$

where fitness differences between species *i* are implemented by assuming differential growth rates *r_i_*. Furthermore, interactions between species *i* and *j*, represented by the coefficient $\alpha_{i,j}$, may differ, and species may have different dispersal rates *λ_i_*. For simplicity, the parameters *r_i_*, *λ_i_*, and $\alpha_{i,j}$ are drawn from normal distributions centered around *r*, *λ*, and *α*, with standard deviations *σ_r_*, $\sigma_{\lambda }$, and $\sigma_{\alpha }$, respectively (negative dispersal rates are set to *λ*). Previous studies (28–31) have shown that without demographic fluctuations and only small differences in interspecies interactions ($\sigma_{\alpha }≲1/\sqrt{S}$), a well-mixed community approaches a unique stationary stable state. On the other hand, when interspecies interaction differs more strongly, well-mixed ecosystems may exhibit multiple (meta-)stable states, which can lead to chaotic dynamics in metacommunities (30, 34).

**Fig. 5.**
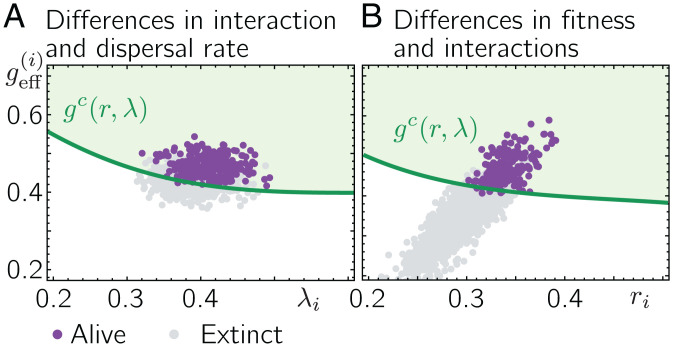
Variation in growth parameters leads to a loss of diversity in species-rich metacommunities. (*A*) Purple and gray circles denote the mean effective growth factors $g_{\text{eff}}^{\left(i\right)}$ of species that are alive or have gone extinct at the end of our numerical solution, respectively. Interaction strengths and dispersal rates are drawn from normal distributions with mean *α* and *λ*, respectively, and standard deviations $\sigma_{\alpha }$ and $\sigma_{\lambda }$, respectively (all growth rates *r_i_* are set to *r*). The green line denotes the extinction threshold value for the growth factor $g_{c}(\lambda )$ (compare with Fig. 2*A*). When the number of competing species is large, the mean effective growth factors of the surviving species cluster close to the threshold value $g_{c}(\lambda )$. (*B*) Fitness *r_i_* and interaction coefficients $\alpha_{i,j}$ are drawn from normal distributions with mean *r* and *α*, respectively, and standard deviations *σ_r_* and $\sigma_{\alpha }$, respectively (all dispersal rates *λ_i_* are set to *λ*). The green line denotes the extinction threshold value for the growth factor as a function of the growth rate *r* [i.e., $g_{c}(r)$]. When the number of competing species is large, the mean effective growth factors $g_{\text{eff}}^{\left(i\right)}$ cluster close to the threshold value $g_{c}(r)$. The remaining parameter values are r=0.3, K=10, λ=0.4, α=0.1, S=300, and P=2,000. For *A*, $\sigma_{\lambda }=0.03$ and $\sigma_{\alpha }=0.5/\sqrt{S}$; for *B*, $\sigma_{r}=0.03,$ and $\sigma_{\alpha }=0.5/\sqrt{S}$. As an initial condition, we chose $N_{x,i}=K$ for all patches and species with small random perturbations. *A* and *B* show the results for three independent draws of parameters and initial conditions.

With the generalized dynamics, Eq. **9**, the effective growth factor of a species *i* at the location *x* is given by $g_{\text{eff}}^{\left(x,i\right)}=1-(r/r_{i})\sum_{j\neq i}\alpha_{i,j}N_{x,j}/K$. When we solve Eq. **9** numerically for short-range dispersal and relatively small parameter differences across species, in particular $\sigma_{\alpha }<1/\sqrt{S}$, we find that the patch-averaged effective growth factors $g_{\text{eff}}^{\left(i\right)}=P^{-1}\sum_{x}g_{\text{eff}}^{\left(x,i\right)}$ initially undergo quick relaxation dynamics followed by weak fluctuations around their steady states. Fig. 5*A* shows the patch-averaged effective growth factors $g_{\text{eff}}^{\left(i\right)}$ after the last time step of our numerical solution of Eq. **9** when only the competition strengths and dispersal rates vary between species ($\sigma_{\alpha },\;\sigma_{\lambda }>0$) and all species have equal fitness (i.e., $\sigma_{r}=0,\;r_{i}=r$). First, we observe that compared with Fig. 2*C*, the patch-averaged effective growth factors $g_{\text{eff}}^{\left(i\right)}$ now vary more strongly between species. Specifically, species also assume patch-averaged effective growth factors that are relatively far above the critical threshold $g^{c}(\lambda )$. A large fraction of species assumes patch-averaged effective growth factors below $g^{c}(\lambda )$ and dies out eventually in the metacommunity (gray circles in Fig. 5*A*). Such a significant number of global extinctions already occurs for relatively small differences in interspecies interactions, where coexistence of most species is supposedly still a stable solution of a well-mixed community coupled to a static mainland (28, 29, 32). Our results hence highlight the important role of self-consistent dispersal in a species-rich metacommunity that is poised at the critical extinction threshold as it is in our case. Considering the surviving species, their patch-averaged effective growth factors (purple circles in Fig. 5*A*) cluster close to the critical extinction threshold $g^{c}(\lambda )$, with the majority of species being close to their (species-specific) extinction thresholds. When allowing differences in fitness and the interaction coefficients (i.e., $\sigma_{r},\;\sigma_{\alpha }>0\;;\;\sigma_{\lambda }=0$), we observe a qualitatively similar behavior in the metacommunity (Fig. 5*B*). While some species assume a patch-averaged effective growth factor relatively far beyond the threshold value $g^{c}(r)$ (plotted as a function of *r* in Fig. 5*B*), others assume a patch-averaged effective growth factor below $g^{c}(r)$ and eventually die out globally. The majority of surviving species assumes patch-averaged effective growth factors close to the threshold $g^{c}(r)$, suggesting that most species operate at their (species-specific) extinction thresholds. Solving Eq. **9** for global dispersal, we find a phenomenology similar to short-range dispersal (see *SI Appendix*, section 8 for a more detailed discussion). In particular, species assume different patch-averaged effective growth factors $g_{\text{eff}}^{\left(i\right)}$ depending on the variances of differential growth rates, dispersal rates, and interaction coefficients (i.e., *σ_r_*, $\sigma_{\lambda }$, and $\sigma_{\alpha }$, respectively). For moderate $\sigma_{\alpha }$, species with $g_{\text{eff}}^{\left(i\right)}$ below the critical threshold *g^c^* eventually go extinct globally, while the $g_{\text{eff}}^{\left(i\right)}$ of the surviving species clusters close above *g^c^*. Increasing $\sigma_{\alpha }$ leads to an increasing spread in patch-averaged growth factors $g_{\text{eff}}^{\left(i\right)}$ (*SI Appendix*, section 8). For large $\sigma_{\alpha }$, we find that some species survive despite having a patch-averaged growth factor $g_{\text{eff}}^{\left(i\right)}$ below the threshold [i.e., $g_{\text{eff}}^{\left(i\right)}<g^{c}$]. We hypothesize that this is related to the existence of multiple stable communities for large $\sigma_{\alpha }$, as suggested in refs. 28, 29, and 32 (see *SI Appendix*, section 8 for a more detailed discussion).

## Discussion

In this study, we have explored the maintenance of biodiversity in closed metacommunities. In contrast to mainland–island models, long-term species survival requires that local species extinction is balanced by dispersal from within the metacommunity. This leads to a minimal dispersal threshold, below which species invariably go extinct. Interestingly, even if dispersal rates exceed this threshold, species tend to self-organize close to an extinction threshold—the more so, the more species are added to the community. The qualitative agreement of our results on both limiting length scales for dispersal (i.e., short-range dispersal and global dispersal) suggests that this self-organization process is a general property of species-rich metacommunities and not restricted to certain length scales of dispersal.

In the case of short-range dispersal, we find that living at the edge of extinction generates fractal spatiotemporal dynamics characteristic of a well-known nonequilibrium phase transition (directed percolation). In contrast to standard directed percolation, this behavior is not restricted to a single point in parameter space (e.g., a critical dispersal rate) but occurs whenever the number of species is large.

The observed convergence of the single-species dynamics to the universality class of directed percolation has important consequences regarding the relevance and applicability of our results to natural ecosystems and alternative theoretical descriptions of metacommunities. First, it suggests that when the number of species is large, all spatiotemporal properties of the single-species dynamics, such as spatiotemporal correlations, the mean survival time, the spreading of a species from a single patch, and the extension of species extinction patterns (compare with Fig. 3), can be described by power laws. Moreover, the exponents of these power laws are all related to each other through merely three critical exponents, all well established by analytical or numerical methods. Second, at least close to the directed percolation threshold, where the mean correlation length and time are expected to diverge, the microscopic details of the underlying model should play a subordinate role for the spatiotemporal dynamics of the system. Our study thus relates the dynamics of species in a species-rich metacommunity to other processes within the broad class of directed percolation, such as models for the spread of epidemics (92), forest fires (93), and range expansions in microbial biofilms (72–74). We furthermore expect that the reported self-organization in species-rich metacommunities and the resulting patterns should be preserved in alternative implementations of metacommunity models with weak competition, including discrete patch occupancy models analogous to Eq. **1**.

While empirical data indicate that spatially averaged static observables, such as abundance distributions, follow rather general trends across different ecosystems (13, 84–86), our study also gives insights into dynamical properties of metacommunities. This allows us to use much more specific spatiotemporal data to verify or falsify our model and to test the proximity of species to global extinction. It will further be interesting to apply our theoretical approach to experiments specifically designed to disentangle colonization and local competition in metacommunities, such as recently proposed with coupled microfluidic chambers (94).

We found that even small variations in the species’ growth, interaction, and dispersal rates lead to extinctions of a fraction of species, where the ability to survive can be characterized by a species’ patch-averaged effective growth factor. This has several implications for the manipulation and preservation of species-rich metacommunities in the view of a changing environment. For instance, environmental perturbations that result in slightly different growth parameters among species (even if transient) can cause a large number of species to go extinct that previously coexisted near their extinction thresholds. In addition, species that are prone to extinction can be saved by selectively increasing their fitness or dispersal rate on some patches so that their patch-averaged effective growth factor falls above the critical extinction threshold. In the course of evolution, we hypothesize that the need for a species to overcome a nonzero critical growth factor to survive may have important consequences for its fixation probability and thus, the evolution of species-rich metacommunities.

In our analyses, we have focused on demographic fluctuations, which are inevitable in a population of discrete individuals. An additional source of noise could stem from environmental (external) fluctuations. Environmental noise is proportional to the number of individuals itself (instead of the square root scaling introduced in Eq. **1**) and has been subject to several previous ecological studies (57, 95, 96). Based on earlier work on metapopulations with environmental noise (97, 98), we hypothesize that in a species-rich metacommunity following Eq. **1** but with environmental noise, species will also be pushed to their extinction threshold, leading to scale-free abundance patterns in the case of short-range dispersal. We expect that the threshold values of the model parameters and the exponents of the resulting power-law distributions will, however, likely be different from the values reported here for demographic noise. In particular, it has been suggested that for metapopulations with environmental noise, the critical growth rate for survival becomes zero in dimensions larger than two when the noise amplitude is below a critical value (97) as well as in the mean-field solution for global dispersal (99).

By considering weak interspecies competition among species where demographic fluctuations dominate the dynamics, our work provides a natural counterpart to several previous studies. These include work on metacommunities with strong fluctuations among patches due to the species’ interactions (34, 47, 58) as well as neutral models (8, 9), which—even in structured metacommunities (13, 100–102)—rely on a continual speciation. Our work underscores the value of self-consistent solutions in coupled ecosystems, which have previously been applied in various contexts, including metapopulations (43, 44, 56, 57) and metacommunities with chaotic dynamics (34, 47). Moreover, our study suggests generalizations of previous work on mainland–island models (28–31) toward closed metacommunities with demographic fluctuations, which we expect to generally feature dispersal thresholds.

## Materials and Methods

### Numerical Solution of the Metacommunity Dynamics.

To numerically solve the metacommunity dynamics described by Eqs. **1**, **4**, and **9**, we employed a numerical update scheme where for every time step Δt, we first calculate the deterministic contributions (i.e., growth, competition, and dispersal of every species on every patch) based on a Euler forward method. After that, demographic fluctuations are implemented by drawing the updated abundances from a Poisson distribution, which ensures the right statistics for the stochastic contributions in Eqs. **1**, **4**, and **9**. All calculations were performed in Python (103), and the results were evaluated using Mathematica (104) (the Python code developed for this study is available at https://github.com/Hallatscheklab/Self-Consistent-Metapopulations). For a more detailed description of the numerical methods, see *SI Appendix*, section 1.

### Mean-Field Theory for Global Dispersal.

For metacommunities with global dispersal, we employed a mean-field theory where the species-averaged and patch-averaged abundances are approximated by their mean-field values $\hat{N}$ and $\bar{N}$, respectively. As detailed in the text and *SI Appendix*, section 5, this mean-field approximation allows us to derive the equilibrium species abundance distribution of Eq. **1**, P, as a function of the mean fields $\hat{N}$ and $\bar{N}$. Finally, we numerically calculate $\hat{N}$ and $\bar{N}$ by demanding self-consistency [i.e., $\hat{N}=\bar{N}={\langle N\rangle }_{P}$, where ${\langle N\rangle }_{P}$ denotes the mean abundance based on the distribution P; all calculations were performed using Mathematica (104)]. From the equilibrium abundance distribution, we can calculate several other equilibrium quantities of the metacommunity, such as the critical dispersal rate *λ_c_*, the critical growth factor *g^c^*, and the mean local diversity (see *SI Appendix*, sections 5 and 6 for details on the derivation and limiting behaviors of these equilibrium quantities). When interspecies competition is weak (0<α≪1) so that multiple species typically coexist on the same patch, our mean-field theory shows very good agreement with our numerical solution of the explicit metacommunity dynamics. For stronger competition between species, such that there are only a few species or even a single species per patch, the numerical explicit solution deviates from our mean-field approximation (*SI Appendix*, section 7).

## Supplementary Material

Supplementary File

## Data Availability

Python code is in GitHub (https://github.com/Hallatscheklab/Self-Consistent-Metapopulations). All other study data are included in the article and/or *SI Appendix*.
