# Supplementary material for: Self-consistent dispersal puts tight constraints on the spatiotemporal organization of species-rich metacommunities
Source: Proc Natl Acad Sci U S A. 2022 Jun 21;119(26):e2200390119. doi: 10.1073/pnas.2200390119 (PMC9245702; doi:10.1073/pnas.2200390119)
Supplement: Supplementary File [file pnas.2200390119.sapp.pdf]

Supplementary Information for

Self-consistent dispersal puts tight constraints on the spatio-temporal organization of species-rich metacommunities

Jonas Denk, Oskar Hallatschek

Corresponding author: Oskar Hallatschek
E-mail: ohallats@berkeley.edu

This PDF file includes:

Supplementary text
Figs. S1 to S6
SI References

Supporting Information Text

1. Numerical solution of the metacommunity dynamics

As detailed in the main text, the metacommunity is assumed to follow the dynamics:

$$\partial_t N_{x,i}(t) = rN_{x,i}\left(1 - \frac{\alpha}{K}\sum_{j,j\neq i}^{S} N_{x,j} - \frac{N_{x,i}}{K}\right) + \sum_{y}^{P}\lambda_{y,x}(N_{y,i} - N_{x,i}) + \sqrt{N_{x,i}}\,\eta\,, \quad [1]$$

where $N_{x,i}$ denotes the abundance of species $i \in \{1,\dots S\}$ on the patch $x \in \{1,\dots,P\}$. The first term denotes population growth and interactions with other species, the second term denotes dispersal between patches, and the third term accounts for demographic fluctuations where η is uncorrelated noise with zero mean and variance ω^2. The square-root dependence of demographic noise on the density ensures that the expected variance of fluctuations is proportional to expected number of birth or death events during one generation and has been derived in various contexts from discrete descriptions of growing populations (1–3). By rescaling the growth rate and the dispersal rate with the rate ω, we measure time in units ω^{-1} and can set $\omega = 1$ in the following. Unless noted otherwise, for Fig. 2-5 in the main text we fixed the growth rate ($r = 0.3$), the competition strength ($\alpha = 0.1$), the carrying capacity ($K = 10$), and solved the dynamics for short-range and global dispersal for various dispersal rates λ and different numbers S of initially coexisting species based on the following Euler forward scheme [all calculations were performed in `Python` (4) and the results were evaluated using `Mathematica` (5)]. For each time step Δt, we first calculate the update of the population size for each species on each patch given by the growth and dispersal dynamics [first two terms in Eq. (1), respectively]. Short-range dispersal is implemented on a one-dimensional lattice with P sites where we set $\lambda_{x,y} = (1/2)\lambda$ for all pairs of neighboring patches x and y and $\lambda_{x,y} = 0$ otherwise (we further assume periodic boundary conditions, i.e. $N_{P+x,i} = N_{x,i}$). For global dispersal, we set $\lambda_{x,y} = \lambda/P$ so that a patch is colonized at a rate $+\lambda\bar{N}_i$, with $\bar{N}_i = P^{-1}\sum_x N_{x,i}$. The contributions from growth and dispersal are calculated and updated separately in order to avoid unphysical scenarios, e.g. that an unoccupied patch acts as a source of dispersal. After updating the deterministic abundance of each species on each patch, demographic fluctuations [last term in Eq. (1)] are added by sampling from a Poisson distribution with the mean being the deterministic abundance. Interpreting Eq. (1) in the Itô sense (6), the Euler forward update for the demographic fluctuations are then incorporated by adding $\sqrt{\Delta t}(\texttt{Poisson}[N_{x,i}] - N_{x,i})$ to the deterministic abundances, where $\texttt{Poisson}[N_{x,i}]$ is a sample from a Poisson distribution with mean $N_{x,i}$. The implementation of demographic fluctuations through a Poisson process guarantees that their variance is given by $N_{x,i}$ (6), consistent with Eq. (1). As initial condition we choose $N_{p,i} = K$ for all patches $x \in \{1,\dots P\}$ and species $i \in \{1,\dots S\}$ with small random fluctuations. Even beyond the onset of finite mean population sizes and in the totally symmetric case, Eq. (1), where all species have identical growth, interaction, and dispersal rates, we observe that a fraction of species goes extinct globally in our numerical solution. We argue that these global extinctions are due to the finite size of our system (finite number of patches P) and should vanish in the thermodynamic limit $P \to \infty$. For the numerical solution of the more general metacommunity given by Eq. 9 in the main text we employ an analogous Euler forward scheme as above where before every numerical solution, the interaction strenghts $\alpha_{i,j}$, the dispersal rates λ_i and the differential growth rates r_i are drawn from normal distributions centered around α, λ, and r, with standard deviations σ_α, σ_λ, and σ_r, respectively (negative dispersal rates are set to λ). For our numerical solutions, the time steps Δt are adapted to values between 0.2 and 1 (when the time step is chosen too large, the update step for the deterministic dynamics can generate negative population sizes, especially when the number of species S is large). The last time step in our numerical solutions ranges between 20000 (Fig. 2, 4, 5) and 50000 (Fig. 3), measured in units ω^{-1}. The Python code developed for this study is available at https://github.com/Hallatscheklab/Self-Consistent-Metapopulations.

2. Distribution of effective growth factors

In the totally symmetric metacommunity, i.e. when the growth rate r, carrying capacity K, inter-species competition strength α, and dispersal rate λ are chosen identical for all species, the effective growth factor for species i on the patch x is defined as

$$g_{\text{eff}}^{(x,i)} := 1 - (\alpha/K)\sum_{j,j\neq i}^{S} N_{x,j}\,. \quad [2]$$

For the parameters chosen in the main text, particularly $0 < \alpha \ll 1$ (e.g. see Fig.2*C*), the patch-averaged growth factors, $g_{\text{eff}}^{(i)} = P^{-1}\sum_{x,i} g_{\text{eff}}^{(x,i)}$, are virtually identical for all species. Fig. S1*A* displays the effective growth factors $g^{(x,i)}$ for all patches for a representative species for different dispersal rates and numbers of species ($S = 5$ and $S = 100$). Furthermore, Fig. S1*B* and *C* show the distributions of effective growth factors $g^{(x,i)}$ for different species and patches at certain dispersal rates close to the extinction threshold (*B*) and farther beyond the extinction threshold (*C*). In general, we find that the distribution of effective growth factors (including the mean) are very similar across different species. Furthermore, the effective growth factors of a species can assume values below the threshold value g^c on individual patches without causing the species' global extinction (Fig. S1*A*). Our results thus suggest that effective growth factors that lie below the extinction threshold on some patches can be balanced by patches with larger effective growth rates and that species can persist in the metacommunity as long as their patch-averaged effective growth factor $g_{\text{eff}}^{(i)} = (P)^{-1}\sum_{x,i} g_{\text{eff}}^{(x,i)}$ exceeds the extinction threshold g^c.

3. Increasing the competition strength can lead to spatial demixing

As discussed in the main text, in this study we focus on scenarios of weak inter-species competitions with $0 < \alpha \ll 1$, so that multiple species can coexist on the same patch. For $0 < \alpha < 1$, the deterministic dynamics of Eq. (1) possesses a stable steady state solution where all species coexist on all patches (Eq. 2 in the main text). In contrast, when $\alpha > 1$, the deterministic dynamics on an isolated patch approaches a steady state with only one species present (7, 8) (referred to as competitive exclusion). This suggests that in a metacommunity with $\alpha > 1$, species may exclude each other on the same patch and occupy different sets of patches (provided dispersal is weak enough to allow heterogeneity between patches), as assumed in previous studies (9–11). In this section we discuss our observation that in the presence of demographic fluctuations a metacommunity can exhibit exclusion of species on the same patch even for $\alpha < 1$. To this end, we increase the competition strength α from zero (i.e. no interaction) to values up to $\alpha > 1$. First, we observe that the mean local diversity generally increases from zero at the threshold $\lambda = \lambda_c$ to larger values with increasing dispersal rate λ (red symbols in Fig. S2*A*). Furthermore, the mean local diversity decreases with increasing α; for large α it falls to values close to and even below one even far beyond the threshold, i.e. for $\lambda \gg \lambda_c$ (e.g. see red triangles for $\alpha = 0.9$ in Fig. S2*A*). The respective kymographs show that in these parameter regimes, species spatially demix and do not interact on most patches (Fig. S2*B*). Moreover, in these regimes of spatial demixing the mean abundance of all species follows the mean abundance of a single species in the absence of interactions. This is seen when plotting the mean abundance of species as a function of the dispersal rate: the bifurcation of the mean abundances indicates a transition between a regime where species demix and behave as if isolated and a regime where species coexist on patches (see squares in Fig. S2*C*).

In the regime where the local diversity is approximately one and species demix, they hardly interact on patches and the definition of an effective growth factor to characterize suppression of population growth by other species is no longer meaningful. We see that in these regimes, the measured patch-averaged growth factors $g_{\text{eff}}^{(i)} = (P)^{-1} \sum_{x,i} g_{\text{eff}}^{(x,i)}$ significantly differs between different species and that $g_{\text{eff}}^{(i)}$ of some species, as well as the the mean effective growth factor $\langle g_{\text{eff}} \rangle = S^{-1} \sum_i g_{\text{eff}}^{(i)}$, can fall below the threshold value g^c (Fig. S2*D*).

We think that this demixing of species for $\alpha < 1$, where the deterministic dynamics alone would yield a spatially mixed metacommunity, poses an interesting question for future studies. As discussed in the main text, in the present work we are, however, particularly interested in scenarios where typically many more than one species coexist on the same patch (i.e. no species demixing) and inter-species competitions are weak, i.e. $0 < \alpha \ll 1$.

4. Characteristic power-law exponents in species extinction patterns

Directed percolation denotes a class of non-equilibrium processes, that describe the spreading of a non-conserved agent through a lattice (12). Implementations of directed percolation, such as the Domany-Kinzel cellular automaton, have frequently been applied to model various spreading processes including forest fires and epidemics [for reviews see (12, 13)], and more recently range expansions of microbial biofilms (14, 15). A continuum description of directed percolation in d dimensions is given by the stochastic differential equation

$$\partial_t \rho(x,t) = a\rho - b\rho^2 + D\nabla^2 \rho + \xi \,, \tag{3}$$

where $\rho(x,t) \geq 0$ is the coarse grained density of the spreading agent (particle density) with space and time variable x and t, respectively, a and b are phenomenological parameters, ∇^2 denotes the diffusion operator in d dimensions with diffusion constant D, and $\xi(x,t)$ denotes a density-dependent Gaussian noise field with the correlations:

$$\langle \xi(x,t) \rangle = 0 \tag{4}$$

$$\langle \xi(x,t)\xi(x',t') \rangle = \Gamma \delta^d(x-x')\delta(t-t')\rho(x,t) \,. \tag{5}$$

With these definitions, the functional form of Eq. (3) is analogous to Eq. 4 in the main text with the discretized diffusion operator $\nabla^2 \to 0.5[\rho(x-\Delta x) + \rho(x+\Delta x) - 2\rho(x,t)]$ for a one-dimensional lattice with lattice constant Δx. Importantly, for an infinite lattice the dynamics Eq. (3) shows a non-equilibrium phase transition (as function of the parameters a, Γ, and D) from an inactive phase, characterized by a density that decays exponentially with time, to an active state, where the density reaches a finite non-zero value. This transition is commonly termed *directed percolation threshold.* Combining field theoretic approaches with numerical solutions of Eq. (3), it was shown that, similarly to a equilibrium phase transition, the critical threshold between the inactive and the active phase is characterized by universal scaling laws for the macroscopic observables including the density. For a finite lattice of extension L the density ρ at the percolation threshold scales as

$$\rho(t,L) \sim t^{-\beta/\nu_\parallel} F(t^{\nu_\perp}/L^{\nu_\parallel}) \,, \tag{6}$$

where F is some function that only depends on the variable combination $t^{\nu_\perp}/L^{\nu_\parallel}$. Here, $\nu_\perp$, $\nu_\parallel$, and β denote the characteristic scaling exponents for directed percolation. Following (16, 17) we can use the scaling Eq. (6) to derive the scaling of the distributions $\mathcal{P}$ for the extensions ℓ and τ of connected unoccupied patches (voids) in space and time, respectively. Assuming the power-law behavior $\mathcal{P}[\ell] \sim \ell^{-\gamma}$ we can write the mean size of connected voids in space within a space interval L in the stationary state as

$$\langle \ell \rangle \sim \int_0^L \mathrm{d}\ell \, \ell \ell^{-\gamma} = L^{2-\gamma} \,. \tag{7}$$

Moreover, from Eq. (6) and the assumption that ρ is finite for large t, we can derive $\langle \ell \rangle \sim 1/\rho(t \to \infty) \sim L^{\beta/\nu_\perp}$. Hence, $\gamma = 2 - \beta/\nu_\perp$. Similarly, assuming the power-law behavior $\mathcal{P}[\tau] \sim \tau^{-\delta}$ we can write the mean size of connected voids in time within a time interval t for a large enough system as

$$\langle \tau \rangle \sim \int_0^t \mathrm{d}\tau\, \tau \tau^{-\delta} = t^{2-\delta}\,, \qquad [8]$$

which, together with the scaling $\langle \tau \rangle \sim 1/\rho(t, L \to \infty) \sim t^{\beta/\nu_\parallel}$ imposed by Eq. (6), yields $\delta = 2 - \beta/\nu_\parallel$. With the values for β, $\nu_\parallel$, and $\nu_\perp$ found for one-dimensional directed percolation (12), we obtain $\gamma \approx 1.747$ and $\delta \approx 1.840$, which is in good agreement with the power laws found for $\mathcal{P}[\ell]$ and $\mathcal{P}[\tau]$ in the species-rich metacommunity (see Fig. 3*C,D*).

5. Self-consistent mean-field approach for global dispersal

In the following we will discuss our analytical mean-field approach to species-rich metacommunities with global dispersal that allows us to calculate static quantities such as the mean population size of species, the abundance distribution and the mean effective growth factor. For convenience, we describe our analysis based on the dynamics for the relative abundances $f_{x,i} = N_{x,i}/K$, given by

$$\partial_t f_{x,i}(t) = r f_{x,i}\left(1 - \alpha \sum_{j,j\neq i}^{S} f_{x,j} - f_{x,i}\right) + \lambda\left[\frac{1}{P}\left(\sum_{y}^{P} f_{y,i}\right) - f_{x,i}\right] + \sqrt{f_{x,i}/K}\,\eta\,. \qquad [9]$$

We introduce the mean fields $\bar{f}_i := P^{-1}\sum_x^P f_{x,i}$ and $\hat{f}_x := S^{-1}\sum_i^S f_{x,i}$, where $\bar{f}_i$ and $\hat{f}_x$ denote the averages of the relative abundance $f_{x,i}$ taken over patches and species, respectively. In the following we assume that the number of patches P and the number of coexisting species on each patch are large, and that all species are statistically identical [with identical growth rates, carrying capacities, interactions and dispersal rates, as in Eq. (9)]. Under this assumption, we estimate the sums over different patches and species in Eq. (9) through the mean field expressions $\bar{f}_i$ and $\hat{f}_x$ and treat these mean fields as deterministic parameters (for a more detailed discussion of the limitations of this approximation see section 7). The dynamics of every species on every patch can then be expressed as

$$\partial_t f(t) = f\frac{\partial \mathbf{F}}{\partial f} + \sqrt{f/K}\,\eta(t) \qquad \text{with:} \qquad \mathbf{F} = rf\left[1 - \frac{f}{2} - \alpha(S-1)\hat{f}\right] + \lambda\left[\bar{f}\log(f) - f\right]\,, \qquad [10]$$

where we omitted the species and patch index since all species and patches are assumed statistically identical. The representation Eq. (10) admits an analytical equilibrium distribution in terms of a Gibbs measure (2, 18–20). To see this, it is convenient to introduce the variable $g := \sqrt{f}$. Using Itô's lemma, we can rewrite Eq. (10) in terms of g as

$$\partial_t g = -\frac{\partial \mathbf{U}}{\partial g} + \frac{1}{2\sqrt{K}}\eta(t) \qquad \text{with:} \qquad \mathbf{U} = -\frac{1}{4}\mathbf{F} + \frac{1}{8K}\log(g)\,. \qquad [11]$$

This dynamics for g can be reinterpreted as the overdamped dynamics of a particle in a potential $\mathbf{U}$ with diffusion constant $1/(4K)$. The equilibrium distribution $\mathcal{P}[g]$ for g is then given through the Gibbs measure

$$\mathcal{P}[g] \sim \mathrm{e}^{-8K\mathbf{U}} = \frac{1}{g}\mathrm{e}^{2K\mathbf{F}}\,, \qquad [12]$$

which is equivalent to a Boltzmann distribution with an "energy" given by $\mathbf{F}$.

In terms of the relative abundance f, we have $\mathcal{P}[f] \propto \frac{1}{f}\mathrm{e}^{2K\mathbf{F}}$, which can be expressed more conveniently as

$$\mathcal{P}[f, \bar{f}, r, K, \alpha, \lambda] = \frac{1}{Z}\frac{1}{f^{1-2K\lambda\bar{f}}}\mathrm{e}^{-Kr[(g_{\text{eff}}-\lambda/r)-f]^2}\,, \qquad [13]$$

where we defined the mean-field effective growth factor $g_{\text{eff}} = 1 - \alpha(S-1)\hat{f}$ and Z denotes the normalization constant. In terms of the abundance $N = Kf$, the distribution can be written as

$$\mathcal{P}[N, \bar{N}, r, K, \lambda] = \frac{1}{Z}\frac{1}{N^{1-2\lambda\bar{N}}}\mathrm{e}^{2rN\left(1-\alpha(S-1)\frac{\hat{N}}{K}-\frac{N}{2K}\right)-2\lambda N}, \qquad [14]$$

with respective normalization Z. Eq. (14). Eq. (13) is the result Eq. 6 in the main text with $\hat{f} = \bar{f} = \bar{N}/K$. While we treated the mean fields $\bar{f}$ and $\hat{f}$ as deterministic parameters, in order for our analysis to be self-consistent they have to be equal and also equal the actual statistical mean of f, which can be calculated from the distribution Eq. (13). Introducing a Lagrange multiplier $+\epsilon f/2K$ into the function F, we can take the derivative of $\log(Z)$ w.r.t to ϵ, take the limit $\epsilon \to 0$, and thereby obtain the mean abundance $\langle f \rangle_{\mathcal{P}[\bar{f},\hat{f},r,K,\alpha,\lambda]}$. Self-consistency then requires:

$$\bar{f} \overset{!}{=} \hat{f} \overset{!}{=} \langle f \rangle_{\mathcal{P}} \qquad [15]$$

Fig. S3*A* shows the calculated mean $\langle f\rangle_{\mathcal{P}}$ as a function of $\bar{f}$ (where for specificity $r=0.3$, $K=10$, $\alpha=0.1$, and $\hat{f}=\bar{f}$ due to self-consistency). All calculations were performed using `Mathematica` (5). Varying the dispersal rate λ we find that for small λ the only solution to the self-consistency condition, Eq. (15), is given by $\bar{f}=0$. Increasing λ above a critical value λ_c, the solution $\bar{f}=0$ is no longer stable; however, there appears a second solution with non-zero $\bar{f}$, which is linearly stable and increases with λ [see Fig. S3*A*]. Thus, λ_c marks a bifurcation from zero to non-zero mean abundances. Expanding the calculated mean $\langle f\rangle_{\mathcal{P}[\bar{f}]}$ to first order in $\bar{f}$, yields the condition for the onset of finite mean abundance:

$$\mathrm{e}^{\frac{(Kr-K\lambda)^2}{Kr}}\frac{K\lambda}{\sqrt{Kr}}\sqrt{\pi}\left(1+\mathrm{Erf}\left[\frac{Kr-K\lambda}{\sqrt{Kr}}\right]\right)\stackrel{!}{=}1\,, \qquad [16]$$

where $\mathrm{Erf}[\cdot]$ denotes the Error-function (incomplete Gaussian integral). Note that the onset of finite mean abundances, Eq. (16), does not depend on the interaction strength α nor the number of interacting species S. This is consistent with the expectation that at the onset of finite mean abundances, interactions between species on a patch should be negligible. As a consequence, Eq. (16) equally describes the onset of non-zero mean abundances in a one-species metacommunity.

Critical dispersal rate For the limiting case $K\lambda \ll Kr$, we expand the condition Eq. (16) up to first order in $K\lambda/(Kr)$ and solved for λ, which yields the critical dispersal rate λ_c:

$$K\lambda_c(r,K)\approx \mathrm{e}^{-Kr}\sqrt{\frac{Kr}{\pi}}\left(1+\mathrm{Erf}[\sqrt{Kr}]\right)^{-1}\,. \qquad [17]$$

When furthermore $K\lambda \ll 1$, the growth rate must be correspondingly large so that we can set $\mathrm{Erf}[\sqrt{Kr}]\approx 1$. This yields $\lambda_c(r,K)\approx \mathrm{e}^{-Kr}\sqrt{\frac{r}{4K\pi}}$, which is Eq. 7 in the main text. The observation of a finite dispersal threshold for global dispersal is consistent with previous studies of metapopulations with implicit spatial extension (21–23), which used master equations to model species birth, death and global dispersal between patches through a shared reservoir. In particular, in the limit $K\lambda \ll Kr$ we recover the same scaling of the critical migration rate with the carrying capacity K as suggested for the large K limit in (22). In the limiting case $K\lambda \gg Kr$ we can expand the condition Eq. (16) to leading order of large $K\lambda/(Kr)$, and solve for λ. This yields the approximation (Eq. 8 in the main text):

$$\lambda_c(r,K)\approx\frac{1}{2K}-r\,, \qquad [18]$$

Hence, we find that for infinitesimally small finite growth rates r, the critical dispersal rate approaches $\lambda_c(r,K)=1/(2K)$. To explore the extreme case $r=0$ for global dispersal, we set the growth rate r equal to zero in the dynamics Eq. (10) and the resulting distribution Eq. (13). Calculating the statistical mean $\langle f\rangle_{\mathcal{P}}$, we find that any mean abundance is marginally stable and fulfills the self-consistency equation Eq. (15), suggesting that the mean abundance is fixed by the initial conditions. This can also be seen by setting $r=0$ in Eq. (10) and averaging, which for global dispersal yields $\partial_t\langle N\rangle=\lambda(\bar{N}-\langle N\rangle)$. Hence, when for instance starting at a homogeneous distribution, $N_{x,i}(t=0)=N_0$, the mean abundance without population dynamics, i.e. $r=0$, will be conserved. Both limiting behaviors of the critical dispersal rate λ_c, at $K\lambda/(Kr)\ll 1$ and $Kr/(K\lambda)\ll 1$, are in very good agreement with respective numerical solutions of the Langevin equation Eq. (9) (see Fig. S4).

Abundance distributions Beyond the onset of finite mean abundances, we can solve for the mean abundance $\bar{f}$ that satisfies the self-consistency condition Eq. (15) numerically [see Fig. S3*B*]. Eventually, substituting this numerical solution for $\bar{f}$ into Eq. (13) yields the equilibrium abundance distribution $\mathcal{P}$ as a function of r, K, α, and the dispersal rate λ. For the parameters used in the main text, including Fig. 4, the bifurcation from zero to finite mean abundances [see Fig. S3*B*] as well as the shape of the abundance distributions calculated by our mean-field approach (see Fig. 4) are in very well agreement with explicit numerical solutions of the full, species-rich dynamics Eq. (9). In section 7 we discuss limitations of the presented mean-field theory and deviations between the mean-field prediction and numerical solutions of the metacommunity dynamics that appear when the mean number of species per patch is small.

Characteristic contributions of the mean-field abundance distribution Above the onset of finite population sizes ($\lambda>\lambda_c$), the abundance distribution derived from our self-consistent mean field approach, Eq. (13), is governed by different contributions, depending on the choice of parameters: When $2\lambda K\bar{f}<1$, the exponent of f in Eq. (13) is negative and the abundance distribution Eq. (13) diverges at zero abundance. The mean relative frequency $\bar{f}$ is limited by $f^*:=N^*/K$, where $N^*=K/[1+\alpha(S-1)]$ denotes the stationary uniform solution of Eq. (1) (see Eq. 2 in main text). Since $f^*\sim(\alpha S)^{-1}$, the abundance distribution Eq. (13) shows a divergence at extinction (i.e. zero abundance) whenever S is large enough, in particular when $S\gtrsim 2K\lambda/\alpha$. When the dispersal rate is small ($\lambda K\bar{f}\ll 1$) and for $f\ll 1$ (i.e. abundances $N\ll K$), the distribution of the relative abundance f follows the scaling

$$\mathcal{P}[f]\propto x^f/f \qquad \text{with:} \qquad x=\mathrm{Exp}[-2K\,(rg_{\mathrm{eff}}-\lambda)]\,. \qquad [19]$$

The form of the abundance distributions $\mathcal{P}[f]\propto x^f/f$ is well-known in ecology literature as Fisher log series (24), which denotes one of the most widely used abundance distributions in ecology and has been recovered in a variety of ecological systems [see (25, 26) for reviews]. Furthermore, the Fisher log series has been derived on mathematical grounds for neutral ecosystems (i.e. where two individuals of the same or different species compete equally, besides having the same growth parameters) with

static immigration as limiting cases for small immigration rates [(27, 28), also see (29) for a review of analytic derivations of abundance distributions for neutral ecosystems]. Thus, our study shows that ecosystems that may exhibit static abundance distributions of neutral ecosystems may in fact underlie quite different species interactions, including weak competition as studied here.

For larger relative abundances ($f \sim 1$), the exponential term in Eq. (13) suggests a local maximum of the abundance distribution characterized by a Gaussian distribution with mean $g_{\text{eff}} - \lambda/r$ and a variance $1/(2Kr)$. When the number of immigrants is small, $2K\lambda\bar{f} \ll 1$, and growth is more likely than dispersal of an individual, $\lambda \ll r g_{\text{eff}}$ the abundance distribution is bimodal, with a contribution for stochastic extinctions at $f = 0$ and a contribution at $f \approx g_{\text{eff}}$ denoting occupied patches. In contrast, farther beyond the onset of finite abundances, $2K\lambda\bar{f} > 1$, the abundance distribution is unimodal, and can be approximated by a Gaussian as mentioned above.

Mean local diversity Another interesting quantity in ecology is the diversity on a single patch (local diversity). The probability of a species on a patch to be extinct, $\mathbf{P}_0$, is the integral over the abundance distribution $\mathcal{P}[N]$, Eq. (14), from zero to one. The mean number of species present on a patch is then given by $d_l = S(1 - \mathbf{P}_0)$. We can find a feasible analytical expression when we assume that the carrying capacity is large ($K \gg 1$) and $\bar{N} \approx N^*$. Then, the abundance distribution Eq. (14) can be approximated by

$$\mathcal{P}[N, \bar{N}, r, K, \lambda] \approx \frac{1}{Z} N^{-1+2\lambda\frac{K}{1+\alpha(S+1)}} \mathrm{e}^{-2\lambda N}, \qquad [20]$$

where Z denotes the respective normalization constant; we further assumed $S \gg 1$ so that $N^*\alpha(S-1) \approx K$. The integral of Eq. (20) from zero to one can be easily calculated and division by the integral of Eq. (20) from zero to infinity eventually yields an approximation for $\mathbf{P}_0$. The respective approximation for d_l is then given by

$$d_l[\alpha, K, \lambda, S] \approx S \frac{\Gamma[\frac{2K\lambda}{1+(S-1)\alpha}, 2\lambda]}{\Gamma[\frac{2K\lambda}{1+(S-1)\alpha}, 0]}, \qquad [21]$$

where $\Gamma[\cdot,\cdot]$ denotes the *Euler Gamma function* defined as $\Gamma[x,y] := \int_y^\infty \mathrm{d}t\, t^{x-1}\mathrm{Exp}[-t]$. In the limit of large S this suggests that the mean local diversity approaches a finite value:

$$d_l \xrightarrow{S\to\infty} 2K\frac{\lambda}{\alpha}\Gamma[0, 2\lambda]\,. \qquad [22]$$

For the parameters chosen in Fig. S5A, we find reasonably good agreement between the estimate Eq. (21) (dashed lines) and the measured mean local diversity in our numerical solution (symbols and solid lines).

6. Critical growth factor and single species percolation threshold

Following the mean-field analysis detailed in section 5, calculating the critical growth factor $g^c(r, K, \lambda)$ for global dispersal is straightforward. The effective one-species dynamics, Eq. 4 in the main text, in terms of the relative abundance $f_x = N_x/K$ for global dispersal can be written as

$$\partial_t f_x(t) = r f_x\,(g - f_x) + \lambda \left[\frac{1}{P}\left(\sum_y^P f_y\right) - f_x\right] + \sqrt{f_x/K}\,\eta\,. \qquad [23]$$

In analogy to section 5, we express the mean over patches through the mean field variable $\bar{f}$, which is then treated as a deterministic parameter. We can then write Eq. (23) as

$$\partial_t f(t) = f\frac{\partial \mathbf{F}}{\partial f} + \sqrt{f/K}\,\eta(t) \qquad \text{with:} \qquad \mathbf{F} = rf\left(g - \frac{f}{2}\right) + \lambda\left(\bar{f}\log(f) - f\right)\,. \qquad [24]$$

This equation has the same form as Eq. 11, allowing us to write the equilibrium distribution of f as a Gibbs measure. From the equilibrium distribution we then obtain an analytic expression for the mean relative abundance $\langle f \rangle$ as a function of the mean field $\bar{f}$. Imposing self-consistency, i.e. $\langle f \rangle = \bar{f}$, eventually yields an expression for the onset of non-zero population size given by

$$\mathrm{e}^{\frac{(g^c\,Kr - K\lambda)^2}{Kr}}\,\frac{K\lambda}{\sqrt{Kr}}\left(1 + \mathrm{Erf}\left[\frac{Kg^c\,r - K\lambda}{\sqrt{Kr}}\right]\right) \overset{!}{=} 1\,, \qquad [25]$$

where g^c denotes the critical growth factor. Eq. (25) can be solved for g^c numerically [see Fig.4A], so that the critical growth factor g^c will be a function of the growth rate r, the carrying capacity K, and the dispersal rate λ.

In the limiting case $K\lambda \ll Kr$ we can expand Eq. (25) up to first order in $K\lambda/(Kr)$ and find the approximation for g^c:

$$g^c \approx \sqrt{\log\left[\frac{Kr}{2K\lambda\sqrt{\pi Kr}}\right]} \Big/ \sqrt{Kr}\,. \qquad [26]$$

To obtain an approximation of g^c in the limit of large dispersal rates, we assume that $g^c Kr \ll K\lambda$, and expand Eq. (25) to first order in $g^c Kr/(K\lambda)$. In this limit, the critical growth factor g^c can be approximated by:

$$g^c \approx \frac{1}{2K\lambda}\,. \qquad [27]$$

7. Limitations of the analytic mean-field approach

As described in section 5, our mean-field approach is based on the assumption that we can express the sums over species and patches in Eq. (9) by their deterministic mean-field values $\hat{f}_x$ and $\bar{f}_i$, respectively. Substituting the sum over patches for the mean-field value $\bar{f}_i$ can be justified by choosing the number of patches P in the numerical solutions sufficiently large. On the other hand, if we express the sum of species on a patch by the deterministic mean-field $\hat{f}_x$, we implicitly assume that the typical number of coexisting species on a patch (local diversity) is sufficiently large. Specifically, the local diversity has to be large enough so that all species on a patch sample a major contribution of the abundance distribution. In the following, we give examples where the mean-field prediction starts to deviate from the numerical solutions as a result of low local diversities.

Mean-field theory deviates from numerical solutions when competition between species increases The deterministic steady state solution $N^* = K/[1 + \alpha(S-1)]$ suggests that the number of species that can coexist (i.e. have an abundance $N_i \geq 1$) on a patch is bounded by $S \leq (K - 1 + \alpha)/\alpha$. Moreover, for $\alpha > 1$, the coexistence state becomes deterministically unstable and an isolated patch will eventually approach a state where only a single species is present (8) (competitive exclusion). While in our study we focus on the scenario of competitions that are so weak that the local diversity is on average much larger than one, in the following we compare scenarios of stronger competitions–and consequently low local diversity–with our mean-field analysis. To this end, we increase α from zero (i.e. no interaction) to $\alpha \gtrsim 1$ and compare our numerical solutions of the explicit multi-species dynamics, Eq. (9) (see section 1), with the predictions from our mean-field theory, section 5.

Mean abundances: Fig. S5*A* shows the mean local and the global diversity (red and blue symbols, respectively) as a function of the dispersal rate for different choices of inter-species competition strength α and initial species S in the metacommunity. Close to the onset of non-zero mean abundance (i.e. $\lambda \gtrsim \lambda_c$) the mean local diversity increases from zero to larger values with increasing dispersal rate. Furthermore, the mean local diversity decreases with increasing α and may, for large α, drop to values around one even for dispersal rates far beyond the onset of non-zero abundance. Especially when the mean local diversity remains small (≈ 1) for dispersal rate beyond the onset λ_c, we find deviations between the mean abundance in our numerical solutions (symbols in Fig. S5*B*) and our mean-field predictions (blue and purple lines Fig. S5*B*). Similar to the case of short-range dispersal (see section 3), we argue that in these parameter regimes species hardly interact with each other on patches but behave like isolated species that occupy only certain subsets of all patches of the metacommunity. In accordance with this conjecture, the mean abundance of our numerical solutions in these regimes approximately follows the mean-field prediction for the mean abundance in the absence of interactions, i.e. $\alpha = 0$ (black dashed lines in Fig. S5*B*). Generally, it appears that the metacommunity assumes states where species are demixed (i.e. the mean local diversity is approximately one) or where multiple species occupy the same patches (i.e. mean local diversity much larger than one), depending on which state yields the larger total abundance for the metacommunity. This observation is reminiscent of our results with short-range dispersal (section 3). We think that the observed demixing of species with short-range and global dispersal, even for $\alpha < 1$, poses an interesting question for future research. In this present study, however, we focus on weak inter-species competition $0 < \alpha \ll 1$, in which case the local diversity is typically larger than one.

Effective growth factors: Similarly to short-range dispersal (section 3), we argue that in regimes where species demix and hardly interact on patches, the definition of an effective growth factor may no longer be meaningful to measure the suppression of population growth due to inter-species competition. As for short-range dispersal, we find that when the mean local diversity drops to values close to one, the patch-averaged effective growth factor differs more between species and the mean effective growth factor can fall below the critical threshold value g^c (symbols in Fig. S5*C* show the species and patch-averaged effective growth factors).

Abundance distributions: In accordance with the observations above, we find that the abundance distributions in our numerical solutions (symbols in Fig. S5*D*) show very well agreement with the mean-field predictions [blue and purple lines in Fig. S5*D*] when the mean local diversity is much larger than one. In regimes of small mean local diversities (≈ 1), the numerical solutions are better described by a mean-field prediction where species do not interact (see black dashed lines in Fig. S5*D*).

In summary, our mean-field approach yields a very good estimate for the metacommunity equilibrium when the local diversity is much larger than one. In particular, we find very good agreement for weak competition strengths ($0 < \alpha \ll 1$), which is the main focus of this work. We find that in parameter regimes with stronger inter-species competitions α (however, already for $\alpha < 1$) and intermediate dispersal rates λ, species can demix due to demographic fluctuations, which leads to deviations from our mean-field predictions. We observe spatial demixing due to demographic fluctuations for both short-range and global dispersal, suggesting that this phenomenon presents a general property of metacommunities and an interesting question for future research. Besides, we also want to mention that, close to the onset of non-zero mean abundances, mean-field theory and numerical solutions are always in very well agreement since there, inter-species interactions are negligible.

8. Variations in growth and dispersal rates of species with global dispersal

To explore more general metacommunities, where species may differ in their growth, interaction, and dispersal rates, we generalized Eq. (1) and consider the following dynamics in the metacommunity:

$$\partial_t N_{x,i}(t) = r_i N_{x,i}\left(1-\frac{N_{x,i}}{K}\right) - r\sum_{j,j\neq i}^{S}\frac{\alpha_{i,j}}{K}N_{x,j} + \sum_{y}^{P}\lambda_{i,y,x}(N_{y,i}-N_{x,i}) + \sqrt{N_{x,i}}\,\eta\,. \qquad [28]$$

Here, fitness differences between species i are implemented by species-specific growth rates r_i. Furthermore, the interaction strengths between species, given by $\alpha_{i,j}$, may differ, and species may have different dispersal rates λ_i. For simplicity, the parameters r_i, λ_i, and $\alpha_{i,j}$ are drawn from normal distributions centered around r, λ, and α, with standard deviations σ_r, σ_λ, and σ_α, respectively (negative dispersal rates are set to λ). With the generalized dynamics Eq. (28), the effective growth factor of a species i at x is given by $g_{\text{eff}}^{(x,i)} = 1-(r/r_i)\sum_{j\neq i}\alpha_{ij}N_{x,j}/K$. For Fig. 5 in the main text we numerically solved Eq. (28) for short-range dispersal and relatively small parameter differences across species, in particular $\sigma_\alpha = 0.5/\sqrt{S}$ and $\sigma_\lambda = 0.03$ (Fig. 5A, main text) and $\sigma_r = 0.03$ (Fig. 5B, main text). Among other things, we find that at the end of our numerical solutions the patch-averaged effective growth factors $g_{\text{eff}}^{(i)} = P^{-1}\sum_x g_{\text{eff}}^{(x,i)}$ of the surviving species cluster close to the critical threshold value g^c.

In the following we will perform a similar analysis for global dispersal and discuss the effect of different magnitudes of growth parameter variations among the species on the metacommunity dynamics in more detail. Previous studies on species-rich, well-mixed communities, that ignored demographic fluctuations (30–33) have shown that when the spread in inter-species interactions σ_α exceeds a certain threshold value σ_α^c (with all species having the same growth rate, i.e. $\sigma_r = 0$), there is no longer a unique stable fixed point of the community. In this regime, a well-mixed community may show multistability (32), while metacommunities with global dispersal have been shown to exhibit spatio-temporal chaos (34, 35). Previous studies of metacommunities (34, 35) typically considered a carrying capacity that very large [$K \sim 10^9$ in (35)] so that demographic fluctuations can be assumed to play a subordinate role for the dynamics. There, extinctions are often implemented by setting population sizes below one individual to zero (*ad hoc* 'cutoff'). Following this implementation for large carrying capacities, in Fig. S6A we increase the spread σ_α of interaction coefficients and observe a transition from a unique stable stationary state to a state of spatio-temporal chaos even when the species growth rates slightly differ ($\sigma_r = 0.01$) *. While in Fig. S6A we increased the variation in inter-species interactions, since this has been the focus for several previous studies of well-mixed ecosystems (30–33), we think it will be similarly interesting to investigate a similar transition when increasing variations in other system parameters such as the growth rates as well as dispersal rates in future studies.

In Fig. S6B we return to a regime of smaller carrying capacities ($K = 1000$) and increase the spread σ_α of interaction coefficients for a metacommunity following Eq. (1), i.e. including demographic fluctuations. When we measure the species' patch-averaged growth factors $g_{\text{eff}}^{(i)} = P^{-1}\sum_x g_{\text{eff}}^{(x,i)}$, we find that initially these undergo quick relaxation dynamics followed by weak fluctuations. As discussed in the main text (Fig. 2C, Fig. 4A, Fig. 5B in main text), increasing the number of competing species S drives the $g_{\text{eff}}^{(i)}$ on average closer to the threshold value g^c. When we increase the spread σ_α in the species' interactions coefficients, the spread of the species' patch-averaged growth factors $g_{\text{eff}}^{(i)}$ grows [circles in Fig. S6B]. For small σ_α [see $\sigma_\alpha = 0.5$, 1 in Fig. S6B], we observe that only species with patch-averaged effective growth factor $g_{\text{eff}}^{(i)}$ above g^c survive (purple circles), while species with smaller $g_{\text{eff}}^{(i)}$ have gone extinct at the end of our numerical solution (gray circles). This is in agreement with our results for short-range dispersal (see section 'Variances in growth parameters lead to stochastic extinctions' in main text and Fig.5). For larger σ_α [see $\sigma_\alpha = 2$ in Fig. S6B], we find that also species with an patch-averaged effective growth factor $g_{\text{eff}}^{(i)}$ smaller than g^c survive. We hypothesize that the ability of species being able to survive despite of below-threshold effective growth factors (i.e. $g_{\text{eff}}^{(i)} < g^c$), is related to the multistability proposed in these regimes of large σ_α [see discussion on multistability above and in Refs (30–33)]. Similar to our discussion in section 3 and section 7, multistability may result in the exclusion among certain communities of species on the same patch and, furthermore, demixing of communities, especially for small dispersal rates. When different communities demix spatially, the effective growth factor averaged over all patches, $g_{\text{eff}}^{(i)}$, may no longer be a good indicator for the survival of individual species. This intuition regarding spatial demixing is further supported when we look at the different communities assumed on different patches at different times. To this end we first identified all different community compositions (i.e. the sets of species that have an abundance of at least one individual) that appear during a numerical solution. Then, we measured how much these communities overlap, i.e. how many species two different communities share. In Fig. S6C we plot the distribution of the relative overlap of different identified communities $\Omega(x,y,t)$, which we define as $\Omega(x,y,t) = |C_{x,t}\cap C_{y,t}|/|C_{x,t}|$ with $C_{x,t}$ denoting all species present at patch x at time t, for 100 time samples and all patches. We find that for relatively small variations in the interaction strengths ($\sigma_\lambda = 0.5$, 1 in Fig. S6C), the distribution of the relative overlap shows a pronounced peak around closely resembling community, i.e. communities with

*The critical threshold value for the transition between a regime with a unique stable stationary fixed point and multistability in a well-mixed community without differences in the species' growth and dispersal rate (i.e. $\sigma_r = \sigma_\lambda = 0$) and uncorrelated interactions is given by $\sigma_\alpha^c = \sqrt{2}(1-\mu)/\sqrt{S} \approx 1.27/\sqrt{S}$ (30–33), where we accounted for the fact, that the interaction strength in our study does not scale inversely with S as in (30–33).

closely resembling species compositions. In contrast, for larger variations in the interaction strengths ($\sigma_\lambda = 2$ in Fig. S6C), there are large contributions from communities with less resemblance (i.e. very different species compositions), suggesting that some communities mutually exclude each other.

We would like to note, that when implementing extinctions by an *ad hoc* 'cutoff' instead of explicit demographic noise even for relatively small carrying capacities [e.g. $K = 1000$ as in Fig. S6B], we observe that species can in general survive with patch-averaged growth factors $g_{\text{eff}}^{(i)}$ below the threshold value g^c [see Fig. S6D]. As discussed in the main text, the need of species to overcome a finite threshold value $g^c > 0$ to survive in our study with explicit demographic noise constitutes an important difference between our work and studies that ignore demographic fluctuations (or implement extinctions by an *ad hoc* 'cutoff'). While g^c approaches zero for large carrying capacities [see Eq. (26), Eq. (27)], we hypothesize that at smaller population sizes, the need to have a patch-averaged effective growth factor beyond g^c can have important consequences, for instance, on the fixation of an upcoming mutant or a species immigrating from outside the metacommunity. Furthermore, for species-rich metacommunities with weak competitive interactions and short-range dispersal, we expect that implementing extinctions through an *ad hoc* 'cutoff' instead of explicit demographic fluctuations will likely generate different spatio-temporal patterns, which lack universal power-law behavior.

References

1. LJ Allen, EJ Allen, A comparison of three different stochastic population models with regard to persistence time. *Theor. Popul. Biol.* **64**, 439–449 (2003).
2. NG Van Kampen, *Stochastic processes in physics and chemistry.* (Elsevier) Vol. 1, (1992).
3. P Leslie, J Gower, The properties of a stochastic model for the predator-prey type of interaction between two species. *Biometrika* **47**, 219–234 (1960).
4. G Van Rossum, FL Drake Jr, *Python reference manual.* (Centrum voor Wiskunde en Informatica Amsterdam), (1995).
5. WR Inc., Mathematica, Version 12.3.1 (2021) Champaign, IL.
6. CW Gardiner, , et al., *Handbook of stochastic methods.* (springer Berlin) Vol. 3, (1985).
7. R MacArthur, R Levins, The limiting similarity, convergence, and divergence of coexisting species. *The american naturalist* **101**, 377–385 (1967).
8. DA Kessler, NM Shnerb, Generalized model of island biodiversity. *Phys. Rev. E - Stat. Nonlinear, Soft Matter Phys.* **91**, 1–11 (2015).
9. RV Solé, D Alonso, A McKane, Self-organized instability in complex ecosystems. *Philos. Transactions Royal Soc. B: Biol. Sci.* **357**, 667–681 (2002).
10. J Mathiesen, N Mitarai, K Sneppen, A Trusina, Ecosystems with mutually exclusive interactions self-organize to a state of high diversity. *Phys. review letters* **107**, 188101 (2011).
11. S Horvát, A Derzsi, Z Néda, A Balog, A spatially explicit model for tropical tree diversity patterns. *J. theoretical biology* **265**, 517–523 (2010).
12. H Hinrichsen, Non-equilibrium critical phenomena and phase transitions into absorbing states. *Adv. Phys.* **49**, 815–958 (2000).
13. G Ódor, Universality classes in nonequilibrium lattice systems. *Rev. modern physics* **76**, 663 (2004).
14. MO Lavrentovich, KS Korolev, DR Nelson, Radial domany-kinzel models with mutation and selection. *Phys. Rev. E* **87**, 012103 (2013).
15. MO Lavrentovich, ME Wahl, DR Nelson, AW Murray, Spatially constrained growth enhances conversional meltdown. *Biophys. journal* **110**, 2800–2808 (2016).
16. R Dickman, MM de Oliveira, Quasi-stationary simulation of the contact process. *Phys. A: Stat. Mech. its Appl.* **357**, 134–141 (2005).
17. G Huber, MH Jensen, K Sneppen, Distributions of self-interactions and voids in (1+ 1)-dimensional directed percolation. *Phys. Rev. E* **52**, R2133 (1995).
18. S Karlin, *A first course in stochastic processes.* (Academic press), (2014).
19. Y Iwasa, Free fitness that always increases in evolution. *J. Theor. Biol.* **135**, 265–281 (1988).
20. NH Barton, HP De Vladar, Statistical mechanics and the evolution of polygenic quantitative traits. *Genetics* **181**, 997–1011 (2009).
21. G Nachman, Effects of demographic parameters on metapopulation size and persistence: an analytical stochastic model. *Oikos* **91**, 51–65 (2000).
22. A Eriksson, F Elías-Wolff, B Mehlig, Metapopulation dynamics on the brink of extinction. *Theor. population biology* **83**, 101–122 (2013).
23. R Casagrandi, M Gatto, A persistence criterion for metapopulations. *Theor. population biology* **61**, 115–125 (2002).
24. RA Fisher, AS Corbet, CB Williams, The Relation Between the Number of Species and the Number of Individuals in a Random Sample of an Animal Population. *The J. Animal Ecol.* **12**, 42 (1943).
25. E Pielou, *Mathematical Ecology.* (Wiley), (1977).
26. GP Patil, EC Pielou, WE Waters, W Waters, WA Waters, *Statistical ecology: spatial patterns and statistical distributions.* (Penn State University Press) Vol. 1, (1971).
27. A McKane, D Alonso, RV Solé, Mean-field stochastic theory for species-rich assembled communities. *Phys. Rev. E - Stat. Physics, Plasmas, Fluids, Relat. Interdiscip. Top.* **62**, 8466–8484 (2000).
28. S Engen, R Lande, Population dynamic models generating species abundance distributions of the gamma type. *J. Theor. Biol.* **178**, 325–331 (1996).
29. RS Etienne, D Alonso, A dispersal-limited sampling theory for species and alleles. *Ecol. Lett.* **8**, 1147–1156 (2005).
30. G Bunin, Ecological communities with Lotka-Volterra dynamics. *Phys. Rev. E* **95**, 1–8 (2017).
31. F Roy, G Biroli, G Bunin, C Cammarota, Numerical implementation of dynamical mean field theory for disordered systems: Application to the lotka–volterra model of ecosystems. *J. Phys. A: Math. Theor.* **52**, 484001 (2019).
32. G Biroli, G Bunin, C Cammarota, Marginally stable equilibria in critical ecosystems. *New J. Phys.* **20** (2018).
33. T Galla, Dynamically evolved community size and stability of random Lotka-Volterra ecosystems(a). *Epl* **123**, 1–13 (2018).
34. F Roy, M Barbier, G Biroli, G Bunin, Complex interactions can create persistent fluctuations in high-diversity ecosystems. *PLoS computational biology* **16**, e1007827 (2020).
35. MT Pearce, A Agarwala, DS Fisher, Stabilization of extensive fine-scale diversity by ecologically driven spatiotemporal chaos. *Proc. Natl. Acad. Sci.* **117**, 14572–14583 (2020).

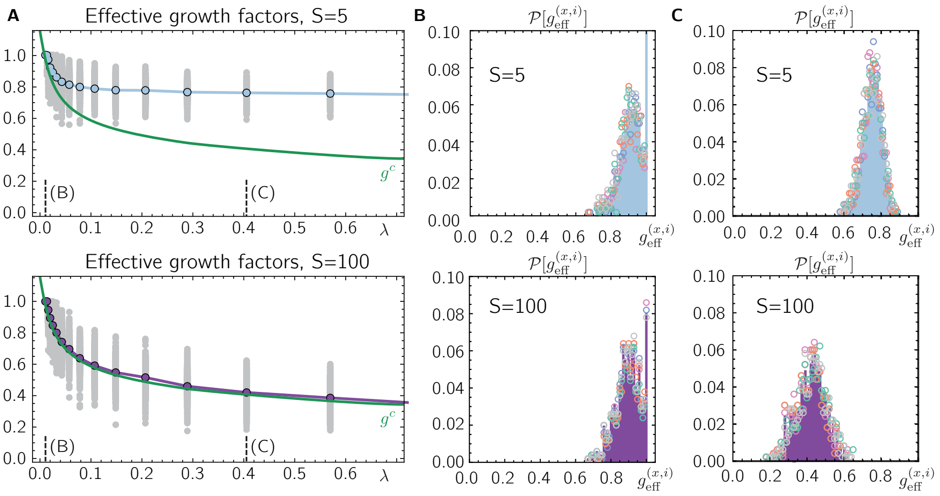

Fig. S1. Distribution of effective growth factors. (A) Effective growth factors $g_{\text{eff}}^{(x,i)}$ of one representative species for different dispersal rates and initially coexisting species $S = 5$ (top) and $S = 100$ (bottom). While individual effective growth factors $g_{\text{eff}}^{(x,i)}$ can fall below the critical extinction threshold g^c (green line), the patch-averaged effective growth factors $g_{\text{eff}}^{(i)} = P^{-1} \sum_x g_{\text{eff}}^{(x,i)}$ (blue and purple circles for $S = 5$ and $S = 100$, respectively) saturate at the extinction threshold g^c for increasing S. **(B), (C)** Distributions of effective growth factors $g_{\text{eff}}^{(x,i)}$ of all species (top: $S = 5$, bottom: $S = 100$) and demes at the final time step of our numerical solution for fixed dispersal rates [*(B)*: $\lambda \approx 0.013$ and *(C)*: $\lambda \approx 0.4$ as indicated by the vertical dashed lines in *(A)*]. Open circles of five different colors denote the distributions of $g_{\text{eff}}^{(x,i)}$ for 5 different species, respectively, which are very similar. Parameters are $r = 0.3$, $K = 10$, $\alpha = 0.1$, $P = 500$.

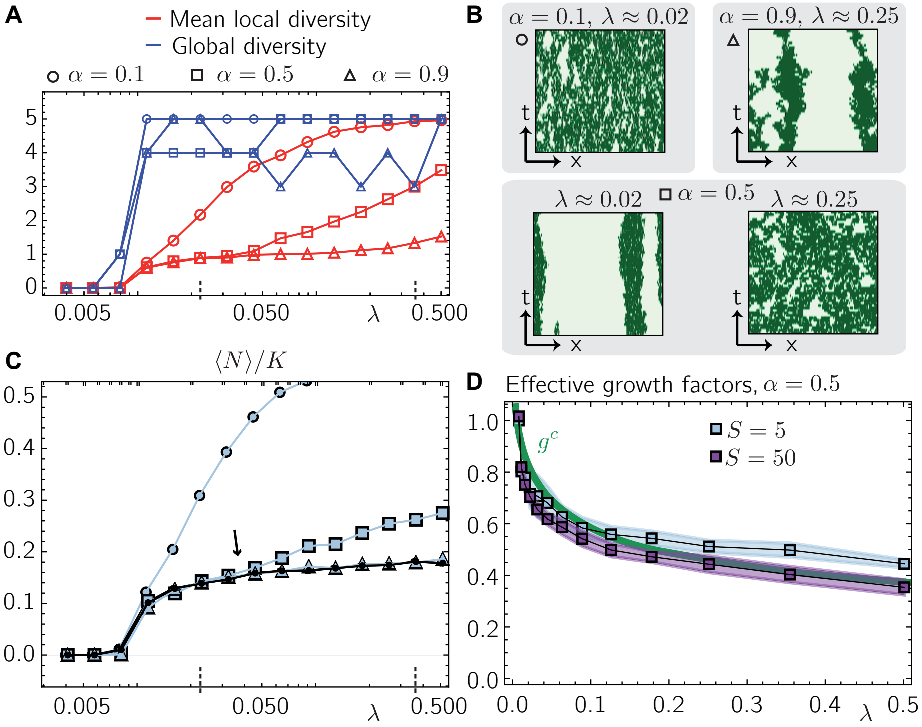

Fig. S2. For larger competition strenghts metacommunities with short-range dispersal show spatial demixing. (A) Total number of surviving species in the metacommunity (global diversity, blue symbols) and mean number of surviving species per patch (local diversity, red symbols) for different inter-species competition strengths α (circles, squares and triangles denote $\alpha = 0.1,\ 0.5$ and 0.9, respectively) and dispersal rates λ for $S = 5$. **(B)** Kymographs of individual representative species show that while for weak competitions, species do not demix (top left panel), stronger competition strenghts α can lead to spatial demixing already when $\alpha < 1$ even for dispersal rates λ far beyond the threshold value λ_c (i.e. for $\lambda \approx 0.25 \gg \lambda_c$, see top right panel). For intermediate competition strengths ($\alpha = 0.5$, lower panels), we observe a transition between spatial demixing for low dispersal rates (left) and no demixing for larger dispersal rates (right). **(C)** Blue shaded symbols denote the mean abundance of all species for different competition strengths α (circles, squares and triangles denote $\alpha = 0.1,\ 0.5$ and 0.9, respectively) as a function of the dispersal rate λ. The black line and circles show the average abundance of a species in the absence of inter-species interactions (i.e. $\alpha = 0$) as a comparison. When the local diversity is close to one [e.g. because of larger α, compare (A)], the mean abundance of species follows the abundance of a single species. Here, species spatially demix and do not do not interact on most patches [as indicated in the kymographs in (B)]. When increasing the dispersal rate λ, for intermediate competition strengths ($\alpha = 0.5$, squares) we observe a crossover between a regime of spatial demixing, where the mean abundance follows the one of non-interacting species, and no demixing, where the mean abundance is larger than the one of non-interacting species (the crossover is highlighted by an arrow). **(C)** Mean (solid lines and squares) and across-species deviations (shaded areas) of the patch-averaged effective growth factors $g_{\text{eff}}^{(i)} = P^{-1}\sum_x g_{\text{eff}}^{(x,i)}$ for $S = 5$ (blue) and $S = 50$ (purple) at intermediate competition strengths $\alpha = 0.5$. The demixing suggested in A-C results in stronger across-species variations of the patch-averaged effective growth factors than for weak competitions (see Fig. 2C in the main text). In the regimes where species demix, we see that the measured patch-averaged effective growth factors can fall below g^c. This effect of demixing is even more pronounced when the number of species is large (e.g. $S = 50$, purple). The remaining parameters are $r = 0.3$, $K = 10$, $P = 500$.

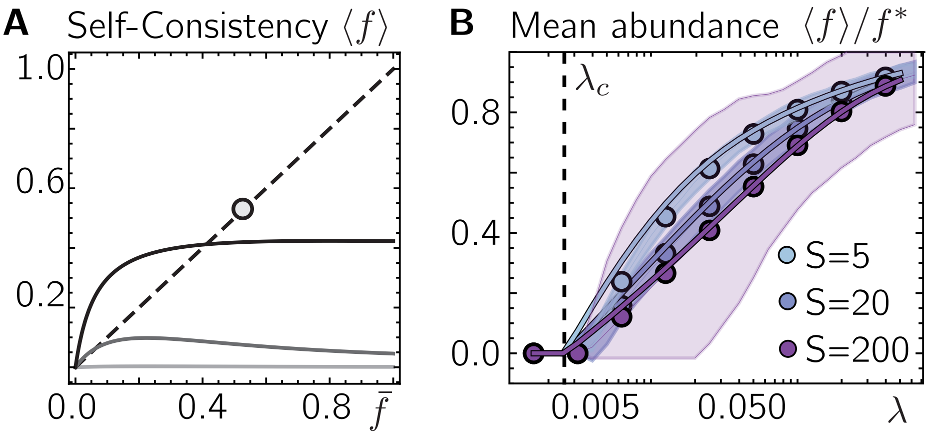

Fig. S3. Self-consistent derivation of the mean abundance of species for global dispersal. **(A)** Above a critical dispersal rate, λ_c, the self-consistency condition $\langle f \rangle = \bar{f}$, Eq. (15) (dashed line), has a solution with non-zero mean abundance $\langle f \rangle$ marking an onset of finite population sizes. Shown are the solutions for $\langle f \rangle$ for dispersal rates smaller, close above and farther above the critical dispersal rate λ_c (from bright to dark grey). The circle denotes the deterministic steady stationary solution $\bar{f} = f^* = N^*/K$. **(B)** The analytic mean field solution for the mean abundance $\langle f \rangle$ (solid lines) is in very good agreement with the numerical solution of the explicit multiple species metacommunity dynamics Eq. (9) (circles). The shaded regions denote the standard deviation of the mean abundances $\langle f_i \rangle$ in our numerical solutions across different species, which can be quite large when the number of species S is large. The parameters are $r = 0.3,\ K = 10,\ \alpha = 0.1$.

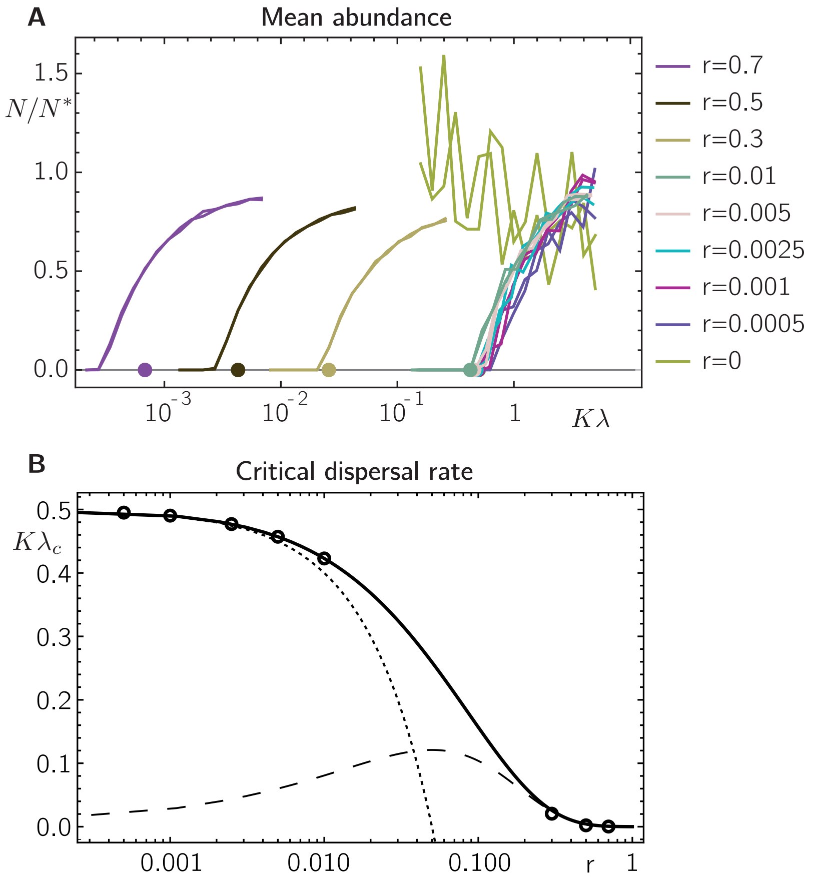

Fig. S4. Limiting behaviors of the critical dispersal rate. (A) When decreasing the growth rate r in our numerical solutions, the transition from zero to non-zero mean abundances N/N^* (solid lines, results for two independent random choices of initial conditions are shown) shifts to larger dispersal rates λ in agreement with our mean-field prediction (circles). As predicted by our mean field theory [see Eq. (18)], our numerical solutions suggests a finite critical dispersal rate λ_c when r approaches 0. At $r = 0$, the final mean abundance of the metacommunity depends more strongly on the initial condition and our numerical results do not suggest a dependence of the mean abundance on the dispersal rate λ. **(B)** The self-consistent mean-field solutions for the critical dispersal rate $\lambda_c(r)$ (solid line) are in very good agreement with our numerical solutions [circles denote the critical dispersal rates obtained from the numerical solutions in ***(A)***]. The dashed and dotted lines denote the limiting behaviors for $K\lambda/(Kr) \ll 1$, Eq. (17), and $Kr/(K\lambda) \ll 1$, Eq. (18), respectively. The remaining parameters are $K = 10$, $\alpha = 0$, and for the numerical solutions: $P = 2000$, Final time: 20000. As initial condition we chose $N_{x,i} = K$ for all patches and species with small random perturbations.

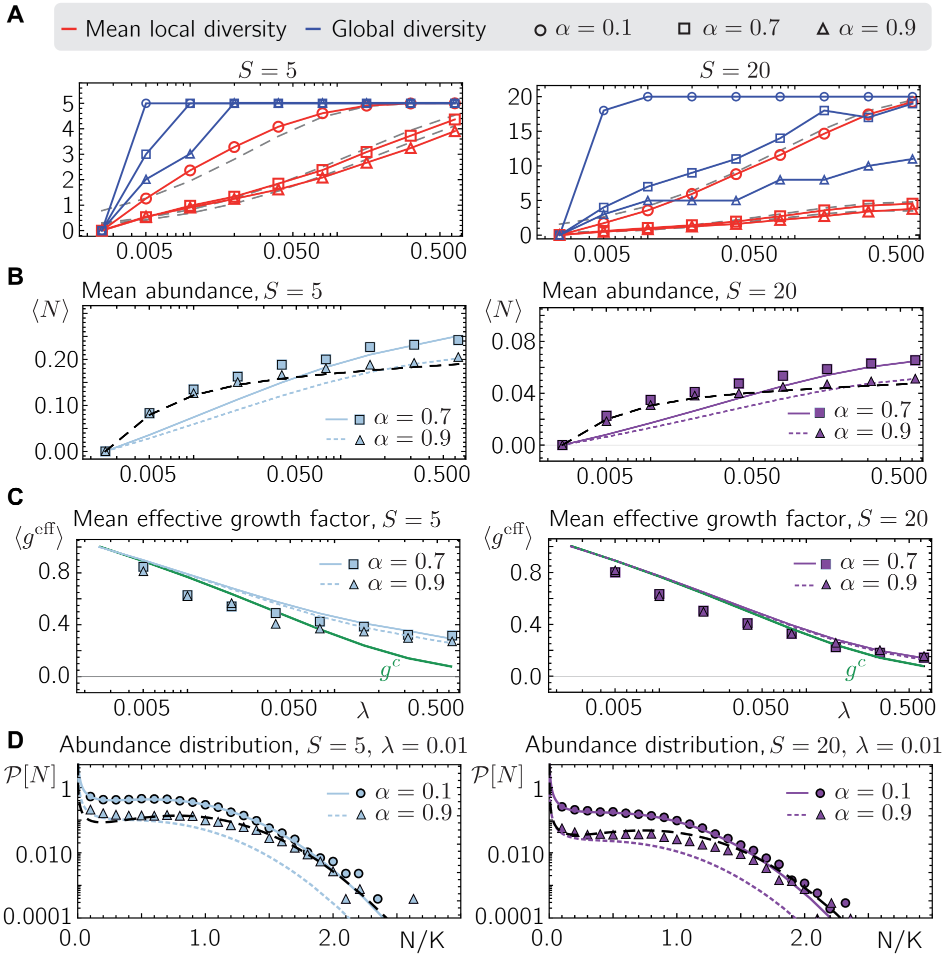

Fig. S5. Mean field theory for increasing competition strengths. (A) Total number of surviving species in the metacommunity (global diversity, blue symbols and lines) and mean number of surviving species per patch (local diversity, red symbols and solid lines) for different inter-species competition strengths α (circles, squares and triangles denote $\alpha = 0.1,\ 0.7$ and 0.9, respectively) and dispersal rates λ for $S = 5$ (left) and $S = 20$ (right). The dashed gray red lines show the mean local diversity according to the approximation $\mathrm{Eq.}\ (21)$. **(B)** Mean-field predictions (colored lines) and the mean abundance of our numerical solutions (symbols) of all species for different competition strengths α (squares and triangles denote $\alpha = 0.7$ and 0.9, respectively) for $S = 5$ (left) and $S = 20$ (right) as a function of the dispersal rate λ. The black dashed line denotes the mean-field prediction for the mean abundance of a species in the absence of inter-species interactions (i.e. $\alpha = 0$) as a comparison. When the local diversity is close to one [e.g. because of larger α, compare (A)], the mean abundance of species (i.e. colored lines) follows the abundance of a single species (i.e. black dashed line). When increasing the dispersal rate λ, for intermediate competition strengths ($\alpha = 0.7$, squares) we observe a crossover between a regime where the mean abundance follows the mean-field prediction for non-interacting species, and a regime where the mean abundance is better approximated by the mean-field prediction for species with competition strength α. **(C)** Mean-field prediction (left: blue lines for $S = 5$, right: purple lines for $S = 20$) and numerical solutions (respective symbols) for the mean patch-averaged effective growth factors $\langle g_{\text{eff}} \rangle = (SP)^{-1} \sum_{x,i} g_{\text{eff}}^{(x,i)}$ at different competition strengths (squares: $\alpha = 0.5$, triangles: $\alpha = 0.9$). In the regimes where species demix (i.e. the local diversity is typically one), we see that the mean patch-averaged effective growth factor can fall below g^c (green solid lines). **(D)** Mean-field prediction (left: blue lines for $S = 5$, right: purple lines for $S = 20$) and numerical solutions (respective symbols) for the abundance distribution. The black dashed lines denotes $\mathcal{P}[N]/S$, the mean-field prediction for the mean abundance of a single species in a metacommunity of non-interacting species. The remaining parameters are $r = 0.3$, $K = 10$, $P = 500$.

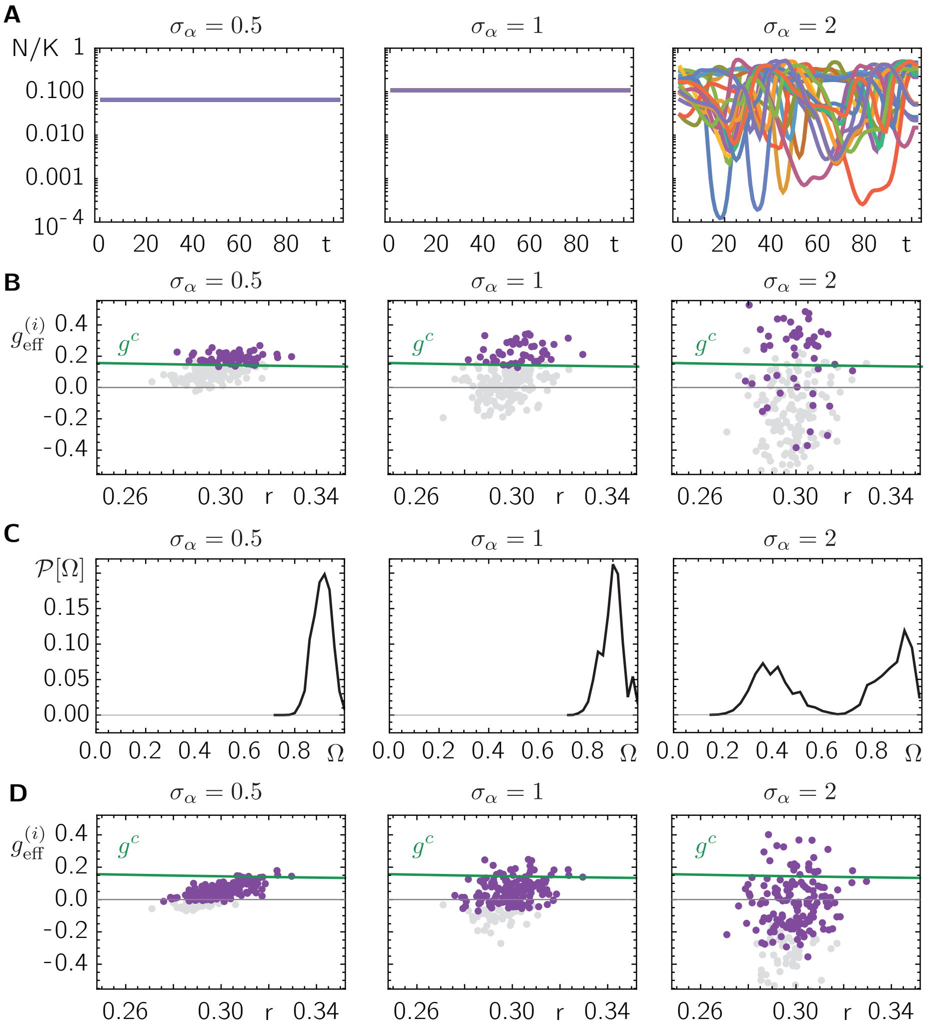

Fig. S6. Variations in growth and interaction parameters lead to species extinctions and clustering close to the extinction threshold. (A) Abundance of one representative species during hundred time steps for different demes (different colors, every second deme is shown). Increasing the spread σ_α leads to a transition between a stationary state to spatio-temporal chaotic dynamics. For these numerical solutions, demographic fluctuations were ignored and an extinction threshold is implemented by setting population sizes of less than one individual to zero (see section 8). $K = 10^9$. **(B)** Patch-averaged effective growth factors $g^{(i)}_{\text{eff}}$ of species at the end of our numerical solution have survived (purple circles) and have gone extinct (gray circles). For σ_α below the transition to multistability indicated in *A*, only species with $g^{(i)}_{\text{eff}} > g^c$ survive, while above the transition also species with $g^{(i)}_{\text{eff}} < g^c$ survive, suggesting that in here $g^{(i)}_{\text{eff}}$ is no longer a good indicator of species survival. $K = 1000$. **(C)** Distribution $\mathcal{P}[\Omega]$ of the overlap Ω (see 8 for definition) of the different communities identified in 100 time samples and all patches. For small σ_α, $\mathcal{P}[\Omega]$ has a sharp peak at closely resembling communities (communities with approx 80% overlap); however, for larger σ_α there are additional large contributions in $\mathcal{P}[\Omega]$ from communities with less resemblance (only approx. 40% overlap), suggesting that some communities mutually exclude each other. **(D)** When extinctions are implemented by an *ad hoc* 'cutoff' (population sizes of less than one individual are set to zero, see section 8) instead of explicit demographic noise, species can survive even when their patch-averaged growth factor is below the threshold value g^c highlighting an important role of demographic fluctuations. For all solutions in *A-D* we used $r = 0.3,\ \alpha = 0.1, \lambda = 10^{-5},\ S = 200$, and $P = 100$.
